# Liganded magnetic nanoparticles for magnetic resonance imaging of α-synuclein

**DOI:** 10.1038/s41531-025-00918-z

**Published:** 2025-04-23

**Authors:** Hope Pan, Melinda Balbirnie, Ke Hou, Naomi S. Sta Maria, Shruti Sahay, Paul Denver, Stefano Lepore, Mychica Jones, Xiaohong Zuo, Chunni Zhu, Hilda Mirbaha, Hedieh Shahpasand-Kroner, Marisa Mekkittikul, Jiahui Lu, Carolyn J. Hu, Xinyi Cheng, Romany Abskharon, Michael R. Sawaya, Christopher K. Williams, Harry V. Vinters, Russell E. Jacobs, Neil G. Harris, Gregory M. Cole, Sally A. Frautschy, David S. Eisenberg

**Affiliations:** 1 Department of Chemistry and Biochemistry, Department of Biological Chemistry, UCLA-DOE Institute, Molecular Biology Institute, UCLA, Los Angeles, CA USA; 2 Department of Research Physiology, Department of Neuroscience, Keck School of Medicine at USC, Los Angeles, CA USA; 3 Geriatric Research Education and Clinical Center, Greater Los Angeles Veterans Affairs Healthcare System, West Los Angeles VA Medical Center, Los Angeles, CA USA; 4 Department of Neurology, David Geffen School of Medicine at UCLA, Los Angeles, CA USA; 5 Department of Neurosurgery, David Geffen School of Medicine at UCLA, Los Angeles, CA USA; 6 Brain Research Institute Electron Microscopy Core Facility, David Geffen School of Medicine, UCLA, Los Angeles, CA USA; 7 Department of Pathology and Laboratory Medicine, David Geffen School of Medicine, UCLA, Los Angeles, CA USA

**Keywords:** Proteins, Neurodegeneration, Parkinson's disease, Neurodegeneration, Magnetic resonance imaging

## Abstract

Aggregation of the protein α-synuclein (α-syn) is the histopathological hallmark of neurodegenerative diseases such as Parkinson’s disease (PD), dementia with Lewy bodies (DLB), and multiple system atrophy (MSA), which are collectively known as synucleinopathies. Currently, patients with synucleinopathies are diagnosed by physical examination and medical history, often at advanced stages of disease. Because synucleinopathies are associated with α-syn aggregates, and α-syn aggregation often precedes onset of symptoms, detecting α-syn aggregates would be a valuable early diagnostic for patients with synucleinopathies. Here, we design a liganded magnetic nanoparticle (LMNP) functionalized with an α-syn-targeting peptide to be used as a magnetic resonance imaging (MRI)-based biomarker for α-syn. Our LMNPs bind to aggregates of α-syn in vitro, cross the blood-brain barrier in mice with mannitol adjuvant, and can be used as an MRI contrast agent to distinguish mice with α-synucleinopathy from age-matched, wild-type control mice in vivo. These results provide evidence for the potential of magnetic nanoparticles that target α-syn for diagnosis of synucleinopathies.

## Introduction

Parkinson’s disease (PD) is a neurodegenerative disease that affects nearly 1 million people in the United States and more than 6 million people worldwide^1^. PD occurs when dopaminergic neurons in the substantia nigra die or lose their function, causing motor symptoms in patients such as bradykinesia, or slowness, tremor, stiffness, and walking and balance problems^2^. In patients of older age or with longer duration of PD, cognitive impairment and dementia can also develop (PD with dementia, or PDD)^3^. Histologically, PD is characterized by the formation of Lewy bodies in neurons of the substantia nigra, composed of the protein α-synuclein (α-syn)^4^. There is a tight correlation between α-syn aggregation and progression of PD^5^, so it has been hypothesized that there is a causative link between α-syn aggregation, its toxicity to dopaminergic neurons, and onset of motor symptoms^6–9^. Aggregation of α-syn is a histological hallmark of other, rarer neurodegenerative diseases, including dementia with Lewy bodies (DLB), in which Lewy bodies are found in neurons similarly to PD^10^, and multiple system atrophy (MSA), in which α-syn aggregates are found in glial cells^11^. Together, these neurodegenerative diseases are termed synucleinopathies^6^.

Currently, patients are diagnosed with PD using a combination of medical history and physical exam findings. Current biomarkers in blood, CSF, or medical imaging do not definitively confirm the diagnosis of PD or measure the progression of disease. Medical nuclear imaging, such as single-photon emission computed tomography (SPECT) and positron emission tomography (PET), can provide additional information for the diagnosis and stage of the disease, as well as biodistribution images of radiotracer targets with high specificity. Clinicians can order a Dopamine Transporter Scan (DaT scan) in which a radio-labeled agent with high affinity for dopamine transporters is administered and detected by SPECT to measure the loss of dopaminergic neurons. However, the DaT scan is not completely predictive of PD and is best used to validate a clinical diagnosis^12^. Recently, progress has been made in pre-clinical development of PET tracers that can track α-syn in vivo^13–17^ and be used as a diagnostic for PD. These nuclear imaging procedures involve ionizing radiation, therefore, the number of times they can be repeated is limited to minimize the effective dose a patient is exposed to. Radiologists and physicians must consider the risks and benefits of performing repeated ionizing procedures to diagnose, stage, and inform the patient^18,19^. Alternatively, magnetic resonance imaging (MRI) is non-ionizing, less expensive, and has greater availability than PET (40.4 MRI scanners vs. 5.5 PET scanners per million people in the United States^20^). Further, MRI can provide high spatial resolution images that cannot be achieved in nuclear imaging.

MRI can take advantage of its high sensitivity to the paramagnetic effects of iron oxide magnetic nanoparticles (MNPs). Iron oxide MNPs create local magnetic field inhomogeneities, shortening the relaxations times measured by MRI (T2 and T2*), and its presence in tissues results in hypointense regions in the images^21,22^. The relaxation rates R2 (1/T2) and R2* (1/T2*) are directly proportional to iron concentration in tissues and have been used to accurately estimate liver and heart concentrations in healthy and disease states^23–25^. Recently, there has been demonstrated potential for liganded iron-oxide nanoparticles to function as an MRI contrast agent for brain metastases^26^ and neurodegenerative disease^27,28^. Iron oxide nanoparticles can cross the blood brain barrier (BBB)^28^, are non-toxic^29^ and non-radioactive, and have sufficient magnetic contrast to be visible by MRI. Iron oxide nanoparticles are already FDA approved and used off-label for MRI^30,31^. Functionalizing magnetic nanoparticles to target alpha-synuclein can potentially provide a non-invasive, highly sensitive, repeatable imaging procedure that is readily translatable to the clinic.

In this study, our goal is to develop a safe and effective diagnostic for PD using an MRI-based agent that targets aggregates of α-syn in the brain. Our agent, which we call liganded magnetic nanoparticles (LMNPs), consists of a dextran-coated iron-oxide nanoparticle conjugated to peptide ligands designed to target α-syn. Our LMNPs bind to aggregates of α-syn in vitro, cross the blood-brain barrier in mice with mannitol adjuvant, and can be used as an MRI contrast agent to distinguish mice with α-synucleinopathy from age-matched, wild-type control mice in vivo. Because formation of α-syn aggregates often precedes the onset of symptomatic PD^6^, this biomarker would enable early detection of PD before clinical symptoms appear. In addition, our MRI contrast agent would be more amenable to longitudinal studies, whereas the amount of exposure to radiation limits the number of PET scans a patient can safely undergo.

## Results

### Rational design of an 11-residue peptide that binds recombinant α-syn

The NACore (^68^GAVVTGVTAVA^78^) is a region of α-syn critical for its aggregation and pathology^32^. In a previous study, we applied computational and structure-based approaches to identify a 24-residue peptide (24mer) composed of GAVVWGVTAV, designed to bind to the NACore, and GRKKRRQRRRPQ, a cell penetrating peptide^33,34^, conjoined by a two lysine linker. 24mer binds recombinant α-syn fibrils and prevents aggregation of recombinant α-syn in vitro^35^.

**Fig. 1:**
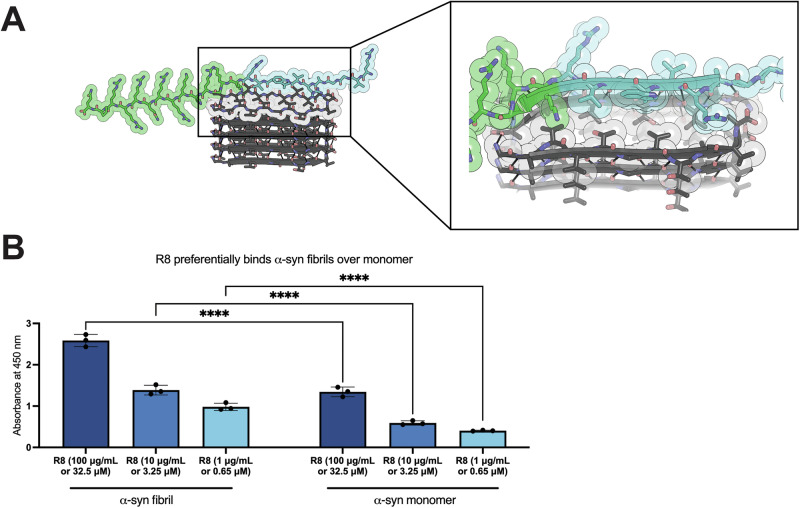
R8 binds recombinant α-syn and prevents its aggregation. **A** R8 is a 25-residue peptide containing an α-syn-binding sequence and a cell penetrating sequence conjoined by a two-lysine linker. The designed binding site of R8 on α-syn is the first part of the NACore and two residues preceding it (^65^NVGGAVVTGVTA^76^). **B** ELISA assesses binding of R8 to α-syn fibril (left) and monomer (right). R8 preferentially binds α-syn fibril over monomer. Statistical analysis was performed using two-way ANOVA (multiple comparisons using Šídák’s multiple comparisons test; ns, *p* > 0.05; *p < 0.05; **p < 0.01; ***p < 0.001; ****p < 0.0001) in GraphPad Prism.

In this study, we sought to pursue a similar approach to generate peptides with greater affinity for binding α-syn. First, we modified the top and bottom strands of the NACore (^68^GAVVTGVTAVA^78^) beta-sheet structure at positions 68, 70, 72, and 74, one at a time and then in pairs. These NACore variants were modified in Coot in CCP4^36^ and then energy minimized in Rosetta^37^ to find potential capping inhibitors. We also looked for peptides that would block the addition of more beta-strands to the N- and C- termini of the beta-sheet by extending the α-syn sequence N-terminally or C-terminally by two residues. In addition, terminal arginines were added to aid in the solubility of the peptide. Through an iterative process, we identified a 25-residue peptide composed of the resulting peptide, RVGGARVWGVR, and GRKKRRQRRRPQ, a cell penetrating peptide, conjoined by a two-lysine linker. We called this peptide R8 (Fig. 1A).

As with 24mer, R8 bound α-syn fibrils. We coated a 96-well plate with α-syn fibrils and monomer and performed an enzyme-linked immunosorbent assay (ELISA) to assess binding of R8 and 24mer at various concentrations. Both R8 and 24mer preferentially bound α-syn fibrils over monomer (Fig. 1B, Supplementary Fig. 1A). When comparing R8 and 24mer binding of α-syn fibrils, R8 bound α-syn fibrils more strongly than 24mer at lower concentrations of 3.55 μM and 0.71 μM, demonstrating that R8 is an improved design for binding α-syn fibrils compared to 24mer (Supplementary Fig. 1B).

Next, we measured the binding affinity of R8 and 24mer for α-syn fibrils using surface plasmon resonance (SPR) (Supplementary Fig. 2). For SPR experiments, we immobilized short, sonicated α-syn fibrils on a CM5 SPR chip and measured fibril-binding affinities of R8 and 24mer at concentrations ranging from 0.3 µM to 10 µM. SPR measurements showed an increase in SPR signal (response units) with an increase in inhibitor concentration for both R8 (Supplementary Fig. 2A) and 24mer (Supplementary Fig. 2B). The equilibrium dissociation constant (K_d_) was calculated by steady state analysis. The apparent K_d_ for R8 and 24mer were determined to be 0.47 µM and 3.7 µM, respectively, demonstrating that R8 was an improved design for binding α-syn fibrils compared to 24mer. These apparent K_d_s are for a single peptide binding to a fibril.

### Liganded nanoparticles bind recombinant α-syn fibrils, DLB brain-derived fibrils, and MSA brain-derived fibrils in vitro

**Fig. 2:**
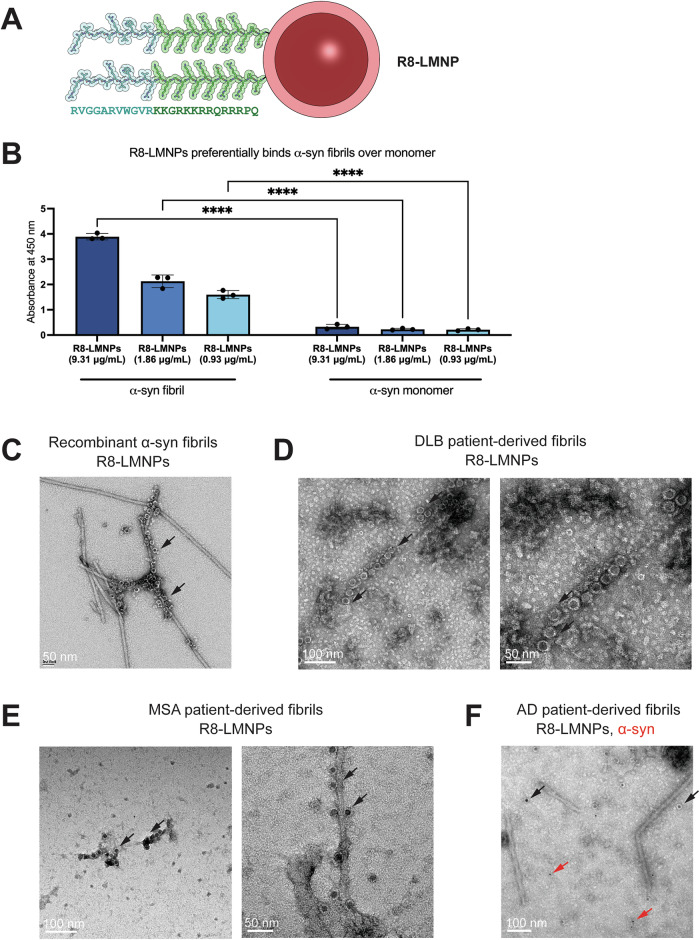
R8-LMNPs bind recombinant, DLB brain-derived, and MSA brain-derived α-syn fibrils. **A** R8-liganded magnetic nanoparticles (R8-LMNPs) are composed of a 10 nm diameter iron oxide core (brown) coated with dextran (pink) covalently coupled to the R8 peptide. Multiple ligands on each nanoparticle promote avidity, enhancing binding. **B** ELISA assesses binding of R8-LMNPs to α-syn fibril (left) and monomer (right). R8-LMNPs preferentially bind α-syn fibril over monomer. Statistical analysis was performed using two-way ANOVA (multiple comparisons using Šídák’s multiple comparisons test; ns, *p* > 0.05; *p < 0.05; **p < 0.01; ***p < 0.001; ****p < 0.0001) in GraphPad Prism. **C** Electron micrograph images of R8-LMNPs (black arrows) binding to recombinant α-syn fibrils. **D** R8-LMNPs (black arrows) bind to DLB brain-derived fibrils. **E** R8-LMNPs (black arrows) bind to MSA brain-derived fibrils. **F** Neither R8-LMNPs (10 nm, black arrows) nor α-syn antibody LB509 (and secondary antibody conjugated to 6 nm gold, red arrows) bind to AD brain-derived fibrils.

We purchased 10 nm, dextran-coated, amine-functionalized iron oxide magnetic nanoparticles (amine MNPs). We used Sulfo-NHS and EDC crosslinking chemistry to couple R8 with the amine MNPs and create R8-liganded magnetic nanoparticles (R8-LMNPs) (Fig. 2A, Supplementary Fig. 3A). Before and after conjugation, amine-functionalized MNPs (Supplementary Fig. 3B) and R8-LMNPs (Supplementary Fig. 3C) were homogenous and well-dispersed. When an equal volume of amine-functionalized MNPs and R8-LMNPs were blotted onto a nitrocellulose membrane, R8-LMNPs spread less far on the nitrocellulose membrane, indicating a change in functionalization of the nanoparticle (Supplementary Fig. 3D). When the membrane was probed with an antibody for dextran, both amine-functionalized MNPs and R8-LMNPs were detected. When the membrane was probed with an antibody for the cell penetrating peptide, only R8-LMNPs were detected, demonstrating successful conjugation of R8 to MNPs (Supplementary Fig. 3D). For comparison, 24mer-LMNPs were constructed following the same protocol.

We first assessed binding of R8-LMNPs and 24mer-LMNPs to recombinant α-syn fibrils. We coated a 96-well plate with α-syn fibrils and monomer and performed an ELISA of R8-LMNPs and 24mer-LMNPs at various concentrations. As for both R8 and 24mer alone, both R8-LMNPs and 24mer-LMNPs preferentially bind α-syn fibrils over monomer (Fig. 2B, Supplementary Fig. 3E). R8-LMNPs bound equally well as 24mer-LMNPs at roughly 70% of the concentration of 24mer-LMNPs, demonstrating that R8-LMNPs bind α-syn fibrils more strongly than 24mer-LMNPs (Fig. 2B). In addition, we attempted to measure the binding affinity of R8-LMNPs and 24mer-LMNPs for α-syn fibrils using SPR. The LMNPs bound so strongly to α-syn fibrils on the SPR chip that they would not dissociate from the chip, so it was not possible to calculate a K_d_ for R8-LMNPs or 24mer-LMNPs. Finally, we incubated recombinant α-syn fibrils (50 μM) with R8-LMNPs (10 μg/mL) for 2 h on ice and visualized them by electron microscopy (Fig. 2C). The LMNPs, which are electron-dense, appeared as dark circles surrounding the negatively stained α-syn fibrils (as indicated by black arrows).

We next assessed R8-LMNP binding to ex vivo brain-derived fibrils. We extracted fibrils from the brains of a patient with dementia with Lewy bodies (DLB) and a patient with multisystem atrophy (MSA) (Supplementary Table 1) and confirmed they were α-syn fibrils by immuno-gold labeling with α-syn antibody LB509 (Supplementary Fig. 4A, B). We incubated DLB brain-derived fibrils or MSA brain-derived fibrils with R8-LMNPs (3 μg/mL) at 4 °C overnight and visualized them by electron microscopy. We observed a mixture of 5 nm and 10 nm width fibrils among both DLB brain-derived fibrils and MSA brain-derived fibrils, consistent with those observed in previous studies^38,39^. R8-LMNPs bound strongly to 5 nm DLB brain-derived fibrils (Fig. 2D) and modestly to 10 nm DLB brain-derived fibrils (Supplementary Fig. 4C). R8-LMNPs bound strongly to both 5 nm and 10 nm MSA brain-derived fibrils (Fig. 2E). Unconjugated, amine-functionalized MNPs did not bind MSA brain-derived fibrils (Supplementary Fig. 4D), demonstrating that α-syn fibril binding by R8-LMNPs is a result of conjugation with the R8 peptide. As a second method to verify R8-LMNP binding, we performed double labeling of DLB brain-derived fibrils or MSA brain-derived fibrils with α-syn antibody LB509 (and secondary antibody conjugated to 6 nm gold) and R8-LMNPs (10 nm iron oxide nanoparticles). We visualized both sizes of nanoparticles binding the DLB brain-derived fibrils (Supplementary Fig. 4E) and MSA brain-derived fibrils (Supplementary Fig. 4F).

Next, we assessed the specificity of R8-LMNPs for α-syn fibrils. We extracted fibrils from the brain of an Alzheimer’s disease (AD) patient (Supplementary Table 1). We performed double labeling of AD brain-derived fibrils with α-syn antibody LB509 (and secondary antibody conjugated to 6 nm gold) and R8-LMNPs (10 nm iron oxide nanoparticles). LB509 did not bind to AD brain-derived fibrils, demonstrating that they were not α-syn fibrils, and R8-LMNPs did not bind fibrils with a paired helical filament morphology, indicating that R8-LMNPs do not bind to tau fibrils (Fig. 2F). To assess whether R8-LMNPs bind amyloid-β fibrils, we performed immuno-gold labeling of AD brain-derived fibrils with amyloid-β antibody D54D2 (Supplementary Fig. 4G). We also performed double labeling of AD brain-derived fibrils with amyloid-β antibody D54D2 (and secondary antibody conjugated to 12 nm gold) and R8-LMNPs (10 nm iron oxide nanoparticles). We visualized both sizes of nanoparticles binding some AD brain-derived fibrils, indicating non-specific binding to amyloid-β (Supplementary Fig. 4H).

### R8-LMNPs cross the blood-brain barrier of M83 mice with mannitol adjuvant

Once we demonstrated that R8-LMNPs could target α-syn fibrils in vitro, we began characterizing R8-LMNP localization in a live mouse model of PD. We chose the M83 model of α-synucleinopathy, which involves transgenic mice expressing the human A53T variant α-syn in central nervous system neurons^40^. By 16 months of age, homozygous M83 mice develop severe motor impairment and widely distributed α-syn inclusions, especially in the spinal cord, brainstem, cerebellum and thalamus. Heterozygous M83 mice develop these symptoms and α-syn inclusions around 22 to 28 months of age^40^.

**Fig. 3:**
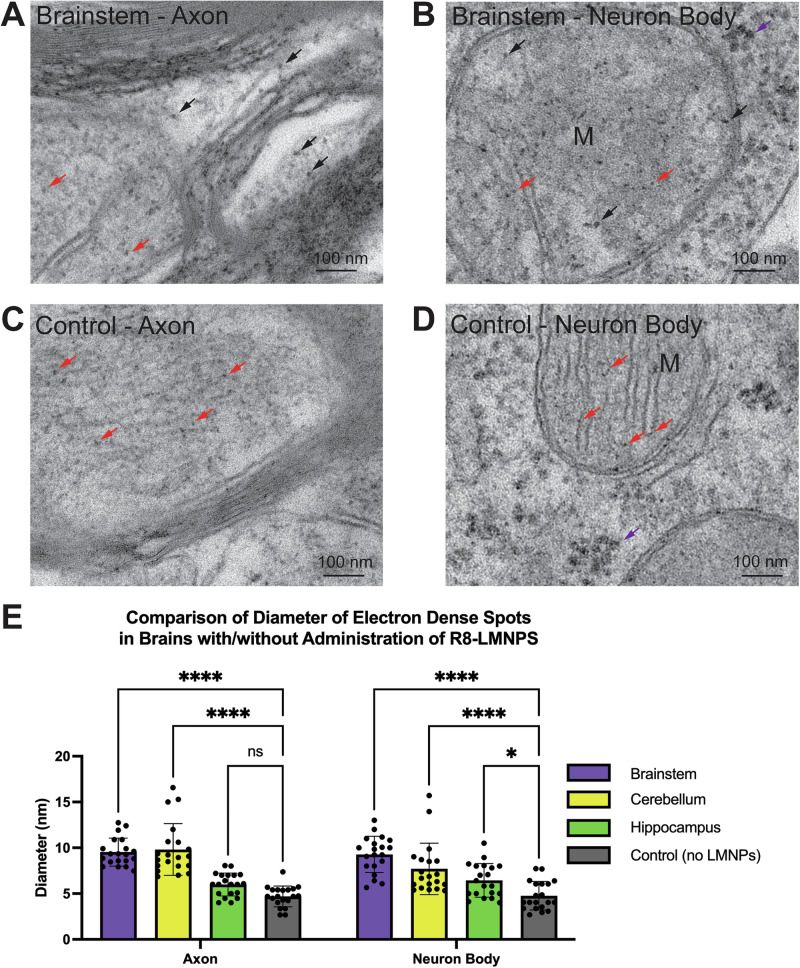
Large electron-dense spots are visible in the brains of M83 mice treated with R8-LMNPs and not in control mice. **A**, **B** R8-LMNPs (10 mg/kg) were administered to aged M83 mice following intranasal administration of mannitol, and mice were euthanized six hours after administration. Images of ultrathin sections of the brainstem were acquired using electron transmission microscopy. Electron-dense spots larger than 10 nm in diameter (black arrows) and smaller than 10 nm in diameter (red arrows) are visible around (**A**) axons and (**B**) neuron cell bodies. These electron-dense spots can be distinguished from ribosomes (purple arrows) in the cytoplasm of the cell body because ribosomes are clustered and about 30 nm in size. Mitochondria are labeled with M. **C**, **D** Mice that did not receive R8-LMNPs were euthanized and their tissue was used as a control. Electron-dense spots are visible around (**C**) axons and (**D**) neuron cell bodies in control tissue, but they are all smaller than 10 nm in diameter. These electron-dense spots can be distinguished from ribosomes (purple arrows) in the cytoplasm of the cell body because ribosomes are clustered and about 30 nm in size. Mitochondria are labeled with M. **E** The average diameter of the electron-dense spots in brains of mice that did not receive R8-LMNPs is significantly lower than the average diameter of electron-dense spots in brains of mice that did receive R8-LMNPs. Statistical analysis was performed using two-way ANOVA (multiple comparisons using Šídák’s multiple comparisons test; ns, *p* > 0.05; *p < 0.05; **p < 0.01; ***p < 0.001; ****p < 0.0001) in GraphPad Prism.

To assess the brain penetration of R8-LMNPs in M83 mice, we administered 24 μL of 2% mannitol intranasally to aid with blood-brain barrier penetration^41^ and immediately administered R8-LMNPs by tail vein injection (10 mg/kg) to two 20-month-old, male, heterozygous M83 mice. After six hours, we euthanized the mice via transcardiac perfusion and collected their brain tissue. The brain tissues were fixed, embedded, and cut into ultrathin sections with an ultramicrotome. The ultrathin sections were put on copper grids, stained with uranyl acetate and lead citrate, and then observed using transmission electron microscopy. In the brainstem of mice that received R8-LMNPs, electron-dense spots were visible near axons and neuron cell bodies (Fig. 3A, B, lower magnification images in Supplementary Fig. 5A). When measured, some electron-dense spots were larger than 10 nm in diameter and others were smaller than 10 nm, with an average diameter between 9.27 nm and 10.99 nm (Supplementary Fig. 5C).

Mice that did not receive administration of R8-LMNPs were also euthanized and their tissue was used as a control. In control tissue, there were also electron-dense spots visible (Fig. 3C, D) near axons and neuron cell bodies (Fig. 3C, D, lower magnification images in Supplementary Fig. 5B). When measured, all electron-dense spots in control tissue were smaller than 10 nm, with an average diameter between 4.69 and 4.76 nm (Supplementary Fig. 5D). The average diameter of the electron-dense spots in brains of mice that did not receive R8-LMNPs is significantly lower than the average diameter of electron-dense spots in brains of mice that did receive R8-LMNPs (Fig. 3E). Because R8-LMNPs were 10 nm in size, we hypothesized that only the electron-dense spots larger than 10 nm in diameter correspond to R8-LMNPs.

In mice that received R8-LMNPs, electron-dense spots were also visible in the cerebellum (Supplementary Fig. 6A, B, lower magnification images in Supplementary Fig. 7A) and hippocampus (Supplementary Fig. 6C, D, lower magnification images in Supplementary Fig. 7B). When measured, some electron-dense spots were larger than 10 nm in diameter and others were smaller than 10 nm, with an average diameter between 7.70 nm and 9.81 nm in the cerebellum, and 5.99 and 6.44 nm in the hippocampus. The number of electron-dense spots larger than 10 nm in diameter varied between brain regions (Fig. 3E). There were many electron-dense spots larger than 10 nm in diameter in the brainstem, less in the cerebellum, and only a few, if not zero, in the hippocampus. These findings correlated with the abundance of α-syn pathology in each brain region, with the brainstem and the cerebellum developing α-syn pathology and the hippocampus developing no α-syn pathology^40^.

In addition, we performed immuno-EM with anti-phosphorylated α-syn antibody pS129 followed by DAB staining and silver enhancement in the brain of an M83 mouse with no administration of R8-LMNPs^40,42^. However, the appearance of the staining was not distinguishable from the appearance of hypothesized R8-LMNPs, making co-localization of α-syn and R8-LMNPs difficult.

### R8-LMNPs remain in brains of M83 mice after 48 h

Next, we assessed the pharmacokinetic properties of R8-LMNPs in M83 mice using inductively coupled plasma mass spectrometry (ICP-MS). We administered R8-LMNPs by tail vein injection (10 mg/kg) immediately following intranasal administration of mannitol to 12-month-old, male, heterozygous M83 mice. We euthanized the mice via transcardiac perfusion at 1 h, 2 h, 4 h, 6 h, 8 h, 24 h, and 48 h of administration (*n* = 3 mice per time point) and collected their brain tissue. We also euthanized and collected brain tissue from 12-month-old, male, heterozygous M83 mice that did not receive R8-LMNPs to serve as a baseline (0 h time point, *n* = 3 mice). We then measured the iron levels in the brain tissue using ICP-MS (Supplementary Fig. 8). Brains from aged M83 mice that did not receive R8-LMNPs were used as a 0 h or “baseline” time point. From one to eight hours after administration of R8-LMNPs, iron levels increased in the brains of M83 mice. At 24 h and 48 h after administration of R8-LMNPs, iron levels plateaued but were still ~50% higher than those at baseline.

### R8-LMNPs can be used as an MRI contrast agent to distinguish M83 mice from age-matched, wild-type control mice

**Fig. 4:**
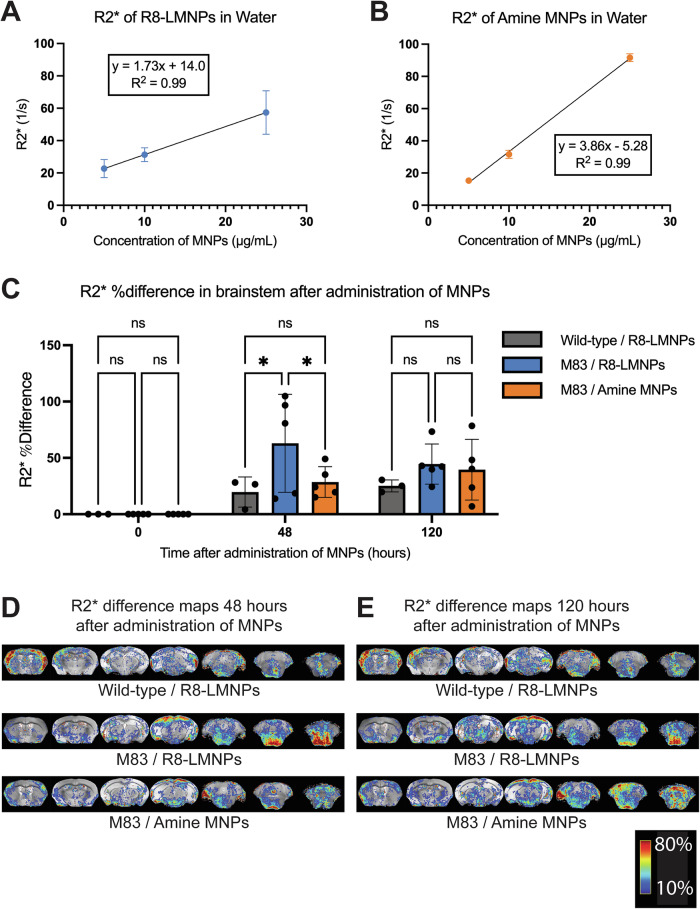
R8-LMNPs can be used as an MRI contrast agent to distinguish M83 mice from age-matched, wild-type controls. R2* relaxation rates of R8-LMNPs (**A**) and amine-functionalized MNPs (**B**) in water. Within the range of 5 to 25 μg/mL, R8-LMNPs and amine-functionalized MNPs cause a linear increase in R2*. **C** Average R2* % difference in the brainstem of M83 mice that received R8-LMNPs (*n* = 5, blue), wild-type control mice that received R8-LMNPs (*n* = 3, gray), and M83 mice that received amine-functionalized MNPs (*n* = 5, orange) either 0, 48, or 120 h after administration of R8-LMNPs or amine MNPs. Statistical analysis was performed using two-way ANOVA (multiple comparisons using Šídák’s multiple comparisons test; ns, *p* > 0.05; *p < 0.05; **p < 0.01; ***p < 0.001; ****p < 0.0001) in GraphPad Prism. Average R2* % difference maps of the brains of M83 mice and age**-**matched wild-type control mice (**D**) 48 or (**E**) 120 h after injection of R8-LMNPs or amine-functionalized MNPs. R2* % difference maps display seven anterior to posterior coronal sections of the brain. R2* % difference maps are colorized on a scale from 0 to 100 ms, with cooler colors representing smaller % increase in R2* and warmer colors representing larger % increase in R2*.

After determining that R8-LMNPs cross the blood-brain barrier in M83 mice with mannitol adjuvant, we sought to characterize their potential use as an MRI contrast agent to distinguish M83 mice from age-matched, wild-type controls. Iron oxide MNPs create local magnetic field inhomogeneities, causing an increase in R2 and R2* relaxation rates on MRI^22,27^. First, we acquired MR images of various concentrations of R8-LMNPs and amine-functionalized MNPs in water on a 7 T MRI (MR Solutions). Within the range of 5 to 25 μg/mL, R8-LMNPs and amine-functionalized MNPs cause a linear increase in R2 (Supplementary Fig. 9A, B) and R2* (Fig. 4A, B) relaxation rates.

Next, we examined whether the increase in R2 or R2* relaxation rate in M83 mice after administration of R8-LMNPs would be significantly greater than the increase in R2 or R2* relaxation rate in wild-type controls. We chose to measure differences in R2 or R2* relaxation rates in the brainstem (defined in Supplementary Fig. 9C), a region of interest (ROI) with abundant α-syn aggregates in aged M83 mice^40^. We administered R8-LMNPs by tail vein injection (10 mg/kg) immediately following intranasal administration of mannitol to one 22-month-old, female, heterozygous M83 mouse and one 22-month-old, female wild-type mouse with the same genetic background (B6C3F1/J). We acquired MR images of their brains at baseline, 8 h, 24 h, 36 h, 48 h, and 120 h post-administration. We observed an increase in the average R2 relaxation rate in the brainstem of both the M83 mouse and the wild-type control from 0 to 48 h (Supplementary Fig. 9D). By 120 h, the average R2 relaxation rate in the brainstem of the wild-type control mouse had returned to baseline levels, whereas the average R2 relaxation rate in the brainstem of the M83 mouse was still elevated (Supplementary Fig. 9D).

To assess whether these differences between M83 mice and wild-type controls would be observed in a larger group, we administered R8-LMNPs by tail vein injection (10 mg/kg) immediately following intranasal administration of mannitol to 22-month-old, female, heterozygous M83 mice (*n* = 5) and age-matched, female wild-type mice (*n* = 3). In addition, we administered amine-functionalized MNPs by tail vein injection (10 mg/kg) immediately following intranasal administration of mannitol to 22-month-old, female, heterozygous M83 mice (*n* = 5). We observed the greatest differences in average R2 relaxation rate between the M83 mouse and the wild-type control mouse in the longitudinal experiment at 48 and 120 h post-administration (Supplementary Fig. 9D); therefore, we acquired MR images of the larger group at baseline, 48 h, and 120 h post-administration.

At 48 h after administration of R8-LMNPs, we observed that the absolute difference (Supplementary Fig. 10) and % difference (Fig. 4C, D, Supplementary Fig. 11) in average R2* relaxation rate in the brainstem of M83 mice were significantly greater than the absolute difference and % difference in average R2* relaxation rate in the brainstem of wild-type control mice. This difference demonstrates that R8-LMNPs can be used as an MRI contrast agent to distinguish M83 mice from age-matched, wild-type controls.

At 48 h after administration of MNPs, the absolute difference (Supplementary Fig. 10) and % difference (Fig. 4C, D, Supplementary Fig. 12) in average R2* relaxation rate in the brainstem of M83 mice that received amine-functionalized MNPs were significantly greater than the % difference in average R2* relaxation rate in the brainstem of M83 mice that received R8-LMNPs. This finding indicates that functionalization with the R8 peptide is required to observe MRI contrast in the brainstem.

In addition, we measured differences in R2* relaxation rate in the hippocampus (defined in Supplementary Fig. 9C), an ROI that does not develop α-syn aggregates in aged M83 mice^40^. At 48 h and 120 h after administration of R8-LMNPs, we did not observe a significant increase in the average R2* relaxation rate in the hippocampus of M83 mice or wild-type control mice (Supplementary Fig. 13). This finding indicates that the significant increase in average R2* relaxation rate is limited to ROIs with abundant α-syn aggregates.

Finally, after MR imaging, we euthanized the mice via transcardiac perfusion and collected and fixed their brain tissue. We performed post-mortem immunohistochemistry on fixed brain tissue sections and observed that there was α-syn pathology in the brainstem, consistent with the increase in average R2* relaxation rate observed by MRI (Supplementary Fig. 14).

### R8-LMNPs do not distinguish 5xFAD mice or PS19 mice from wild-type control mice

To investigate whether R8-LMNPs bind to tau or amyloid-β aggregates in vivo, we assessed whether R8-LMNPs can distinguish 5xFAD mice and PS19 mice from wild-type control mice. 5xFAD mice develop amyloid-β pathology in the deep cortex and subiculum as early as two months of age^43^. PS19 mice develop hyperphosphorylated tau inclusions after six months of age^44^, and young PS19 mice can be seeded by intracranial injection of tau fibrils to rapidly induce tau pathology^45,46^. We administered R8-LMNPs by tail vein injection (10 mg/kg) immediately following intranasal administration of mannitol to 5.5-month-old, male, heterozygous 5xFAD mice (*n* = 3) and 5-month-old, male, heterozygous PS19 mice seeded with AD brain-extracted tau fibrils in the left and right hippocampus (*n* = 4). We acquired MR images at baseline, 48 h, and 120 h post-administration.

At 48 h and 120 h after administration of R8-LMNPs, we chose to measure average R2* relaxation rate in the cortex, an ROI with abundant amyloid-β pathology in 5xFAD mice. We observed an increase in average R2* relaxation rate in the cortex of 5xFAD mice, indicating possible non-specific binding of R8-LMNPs to amyloid-β (Supplementary Fig. 15A, B). However, the increase in average R2* relaxation rate was not significantly greater than the increase in average R2* relaxation rate in the cortex of wild-type control mice (Supplementary Fig. 15C).

At 48 h and 120 h after administration of R8-LMNPs, we chose to measure average R2* relaxation rate in the hippocampus, the ROI where PS19 mice were seeded with AD tau. We observed no significant increase in average R2* relaxation rate in the hippocampus of AD brain-seeded PS19 mice compared to wild-type control mice, indicating no non-specific binding of R8-LMNPs to tau (Supplementary Fig. 16).

## Discussion

Aggregation of the protein α-synuclein (α-syn) is a histological hallmark of multiple neurodegenerative diseases collectively known as synucleinopathies, including Parkinson’s disease (PD), PD with dementia (PDD), dementia with Lewy bodies (DLB), and multiple system atrophy (MSA)^4,10,11^. In this study, our goal was to develop a liganded magnetic nanoparticle (LMNP) that could target fibrillar aggregates of α-syn in the brain and be used as an MRI contrast agent for the diagnosis of PD and other synucleinopathies.

We identified a 25-residue peptide, which we termed R8, composed of an α-syn targeting peptide and a cell penetrating peptide, conjoined by a two-lysine linker. R8 preferentially binds to α-syn fibrils over monomers (Fig. 1), and the apparent K_d_ for R8 binding to α-syn fibrils was measured as 0.47 µM (Supplementary Fig. 2). We coupled R8 with the amine-functionalized magnetic nanoparticles (MNPs) to create R8-liganded magnetic nanoparticles (R8-LMNPs). R8-LMNPs preferentially bind to α-syn fibrils over monomers and R8-LMNPs bind to recombinant α-syn fibrils, DLB brain-derived fibrils, and MSA brain-derived fibrils (Fig. 2).

We administered R8-LMNPs by tail vein injection following intranasal administration of mannitol to aged M83 mice, which are known to develop pathology in the spinal cord, brainstem, cerebellum, and thalamus. We observed electron-dense, 10 nm-large R8-LMNPs in the brains of M83 mice (Fig. 3), and we observed that R8-LMNPs remain in the brains of M83 mice for at least 48 h (Supplementary Fig. 9). Finally, we administered R8-LMNPs by tail vein injection to M83 mice and age-matched, wild-type control mice and observed that the increase in average R2* relaxation rate in the brainstem of M83 mice was significantly greater than the increase in average R2* relaxation rate in the brainstem of wild-type control mice (Fig. 4). These findings demonstrate that R8-LMNPs can distinguish M83 mice from age-matched, wild-type controls by MRI in vivo and could potentially distinguish human patients with α-syn pathology from healthy human controls by MRI.

Recent studies have shown that recombinant α-syn fibrils, α-syn fibrils found in patients with MSA, and α-syn fibrils found in patients with PD/PDD/DLB all have different structural polymorphs^47–49^. We demonstrated that R8-LMNPs can bind to recombinant α-syn fibrils, DLB brain-derived fibrils, and MSA brain-derived fibrils, so it is unknown whether they distinguish between polymorphs of α-syn fibrils. However, it is possible that R8-LMNPs could distinguish between different synucleinopathies by MRI because of the differing distribution of α-syn aggregates. In PD, Lewy bodies are mostly found in the substantia nigra^4^, whereas in DLB, Lewy bodies are found in both the neocortex and substantia nigra^10^. In MSA, glial α-syn aggregates are found in the substantia nigra, the brainstem, and the cerebellum^11^. These differing distributions, paired with the knowledge that synucleinopathies feature distinct clinical features^2,3,50–54^, may still allow for R8-LMNPs to distinguish between synucleinopathies.

There are some limitations to this study. First, R8 binds to α-syn fibrils with an apparent K_d_ of 0.47 µM, which is weaker than the binding affinity of α-syn PET ligands being investigated^13–17^. However, the nanoparticles have multiple sites for peptide conjugation. Therefore, each R8-LMNP is a multivalent peptide scaffold, increasing the binding affinity through avidity, as demonstrated by SPR experiments in which LMNPs did not release α-syn fibrils on the SPR chip. In addition, the lower binding affinity of R8 may be compensated by the iron oxide nanoparticles’ paramagnetic properties that are detected by MRI with high sensitivity^26^. If needed, the binding affinity of R8 could be improved with affinity maturation using peptide display methods^55^. Secondly, we observed non-specific binding of R8-LMNPs to amyloid-β in vitro. This result is unsurprising due to the sequence similarity between amyloid-β and the non-amyloid component region of α-syn. However, R8-LMNPs did not distinguish 5xFAD mice from control mice by MRI, so it is unclear whether R8-LMNPs have non-specific binding to amyloid-β in vivo. Finally, it has been established that mouse models of α-synucleinopathy and other amyloidopathies do not completely replicate human pathology^56^. Although our results demonstrated that R8-LMNPs can distinguish M83 mice from age-matched, wild-type controls by MRI, it is possible that they may not target α-syn pathology and be useful as a contrast agent in humans. However, our finding that R8-LMNPs can bind to DLB brain-extracted fibrils and MSA brain-extracted fibrils in vitro suggest promise for in vivo binding as well.

Before R8-LMNPs could be considered for use in humans, additional studies would need to be done. First, it remains to be determined whether mannitol is necessary for effective BBB penetration of R8-LMNPs. Previous studies using similar iron oxide MNPs as MRI contrast agents have shown that MNPs can cross the BBB without the use of mannitol^28^. However, if further testing reveals that mannitol is necessary for BBB penetration of R8-LMNPs, R8-LMNPs and mannitol could be co-administered intravenously, similarly to the mannitol in the FDA-approved drug Feraheme, which is delivered by intravenous infusion^30^. Secondly, our aim was to develop a LMNP that could be used as a contrast agent for early diagnosis of α-synucleinopathies; however, we assessed R8-LMNPs in aged M83 mice with abundant pathology. In future studies, we will assess whether R8-LMNPs increase R2* relaxation rates in younger M83 mice with less pathology to better characterize the sensitivity of R8-LMNPs for early stages of pathology. Finally, additional pharmacokinetic and pharmacodynamic studies will be needed to better characterize the absorption, distribution, metabolism, and excretion (ADME) of R8-LMNPs. We determined that R8-LMNPs remain in the brain of M83 mice for at least 120 h. However, it remains unclear when they are cleared from the brain, which would affect the frequency of repeat dosing in longitudinal studies. In addition, it remains unclear whether R8-LMNPs are metabolized after administration, which would affect their ability to target α-syn pathology in the brain.

In summary, R8-LMNPs bind to recombinant, DLB brain-derived, and MSA brain-derived fibrils in vitro, cross the blood-brain barrier in M83 mice with mannitol adjuvant, and can be used as an MRI contrast agent to distinguish M83 mice from age-matched, wild-type controls in vivo. These results provide evidence for the potential of using liganded magnetic nanoparticles that target α-syn for the diagnosis of PD and other synucleinopathies.

## Methods

### Expression and purification of recombinant α-syn

Recombinant α-syn was expressed and purified as previously described^35^. The final step in the purification was HPLC Size exclusion chromatography on a preparative G3000 column (Tosoh Bioscience). The resulting α-syn monomer was flash frozen in small aliquots for subsequent experiments.

### Generation of recombinant α-syn fibrils

Human recombinant α-syn fibrils were grown at 400 μM in PBS at 37 °C, shaking in a Torrey Pines shaker, level 9, over three days. Fibril formation was confirmed by taking a small aliquot, adding thioflavin T, plating in a 96-well plate and verifying fluorescence with the SpectraMax M5 plate reader (excitation wavelength of 444 nm and emission wavelength of 485 nm). Additionally, the presence of fibrils was verified by transmission electron microscopy by diluting the sample to 10 μM and spotting on a glow-discharged carbon-formvar grid, and imaging, as described below.

### Peptide ELISA

Recombinant α-syn monomer and fibrils were plated at 3.5 μM (100 μL/well) in a high-binding ELISA plate (Greiner, 655061) and placed at 4 °C overnight. The next day, wells were blocked with Superblock T20 (Thermo Fisher Scientific), washed with TBS-T (all TBS-T washes were three times for 5 min each), and then incubated with R8 and 24mer peptides in PBS (all concentrations in triplicate) for 2 h at room temperature. After washing with TBS-T, wells were incubated with primary antibody mouse N3 anti-HIV-1 tat (Abcam) at a 1:300 dilution for one hour. Wells were washed with TBS-T and then incubated with Goat anti-mouse HRP (Sigma) diluted 1:5000 for one hour. After washing with TBS-T, wells were treated with 100 μL room temperature TMB-ELISA and left to develop a blue color. Reactions were quenched with 2 M HCl and absorbance at 450 nm was read on a Spectramax M5 plate reader (Molecular Devices). Monomer and fibril wells without added peptide were probed with an anti-α-syn antibody (BD Transduction Labs, 610787) as a surrogate loading control.

### Surface plasmon resonance

SPR experiments were carried out on a BiaCore T200 instrument (GE Healthcare). For preparation of the CM5 conjugated SPR chip (Cytiva), fibrils were centrifuged at 15,000 rpm at 4 °C for 45 min, and the pellet was resuspended in PBS in a volume equivalent to the removed supernatant. The fibrils were sonicated, filtered, and concentrated prior to conjugation to the CM5 chip. Peptides were diluted in PBS to concentrations ranging from 300 nM to 10 μM. For R8 and 24-mer peptides, all concentrations were recorded in triplicate. R8 data were collected with a 60 s contact time, 24-mer data were collected with both 30 and 60 s contact times. Using the steady state analysis and a 1:1 binding model, the apparent Kd was determined.

### Synthesis of R8-LMNPs

Amine-functionalized iron oxide nanoparticles, 10 nm in size, were purchased from Creative Diagnostics in New York, USA (WNM-X008). R8 peptide was synthesized by Genscript (Piscataway, NJ) or LifeTein (Somerset, NJ). 0.5 mg of R8 peptide was dissolved in 100 μL ultrapure Millipore water and filtered through a 0.22-micron filter. 2 mg of sulfo-NHS and 0.32 mg of EDC were dissolved in 100 μL activation buffer (0.1 M MES, 0.5 M NaCl, pH 6.0), respectively, before they were mixed. 100 μL sulfo-NHS/EDC mix was added to the 100 μL peptide solution and kept stirred at room temperature for 15 min. Next, 200 µl of the R8/EDC/NHS solution was mixed with 100 µl 20X PBS and 100 µl 5 mg/ml MNP and incubated at room temperature for 2 h. After 2 h, the solution was kept in a magnetic separator at 4 °C overnight. The next day, the supernatant was removed from the nanoparticles, which were adhered to the wall of the Eppendorf tube in the magnetic separator. The nanoparticles were re-dissolved in 100 µl PBS and the magnetic separation was repeated three times to wash the nanoparticles. Finally, the nanoparticles were re-dissolved in 100 µl PBS and stored at 4 °C.

### Dot blot of amine-functionalized MNPs and R8-LMNPs

1.5 μL of amine-functionalized MNPs and R8-LMNPs were blotted onto two nitrocellulose membranes. The membranes were blocked in 5% milk in TBST for 45 min. One membrane was probed with an antibody for dextran (DX1, STEMCELL Technologies) in 2% milk in TBST (1:500) for 2 h. The other membrane was probed with an antibody for the Tat peptide (N3, Invitrogen) in 2% milk in TBST (1:500) for 2 h. The membranes were washed three times with TBST and then probed with an HRP-labeled secondary antibody in 2% milk in TBST (1:5000) for 1 h. The membranes were washed three times with TBST and then labeled with Thermo Scientific Pierce ECL Western Blotting Substrate. The dot blot was imaged with an Azure Biosystems imaging system.

### Transmission electron microscopy of recombinant fibrils and R8-LMNPs

Recombinant α-syn fibrils (50 μM) were mixed with R8 peptide-conjugated iron oxide magnetic nanoparticles (10 μg/mL) on ice for 2 h and then spotted on freshly glow discharged 400 mesh carbon formvar grids (Ted Pella, Inc. 01754-F) at a final concentration of 10 μM α-syn. Grids were washed once in water and then stained for 3 min with 2% uranyl acetate, followed by a second water wash, and then allowed to dry. Grids were imaged at 6800x to 49,000x magnification on a FEI Tecnai Electron Microscope operating at 120 kV.

### Extraction of fibrils from DLB brain tissue

Fresh-frozen brain regions of individuals with DLB were extracted using protocols adapted from Schweighauser et al.^48^. Briefly, tissues were homogenized in 15 vol (v/w) extraction buffer consisting of 10 mM Tris-HCl, pH 7.5, 0.8 M NaCl, 10% sucrose and 1 mM EGTA. Homogenates were brought to 2% sarkosyl and incubated for 30 min at 37 °C. Following a 10 min centrifugation at 10,000 *g*, the supernatants were spun at 100,000 *g* for 22 min. The pellets were resuspended in 1 ml/g extraction buffer and centrifuged at 3000 *g* for 5 min. The supernatants were diluted threefold in 50 mM Tris-HCl, pH 7.5, containing 0.15 M NaCl, 10% sucrose and 0.2% sarkosyl, and spun at 166,000 g for 32 min. Sarkosyl-insoluble pellets were resuspended in 250 μl/g of 30 mM Tris-HCl, pH 7.4.

### Extraction of fibrils from MSA brain tissue

Extraction of sarkosyl-insoluble α-syn fibrils from neuropathologically confirmed brain samples of patients diagnosed with MSA was performed using the method previously described by Schweighauser et al. without any modifications^48^.

### Immunogold labeling of DLB or MSA brain-derived fibrils with α-syn antibody

Fibrils were spotted on a freshly glow discharged carbon formvar 400 mesh grid (Ted Pella, Inc.), blotted and then blocked in 0.1% gelatin in PBS for 1 h at room temp. Grids were then floated on a 50 μL drop of a 1:100 dilution of LB509 (Santa Cruz Biotechnology, sc-58480) in 0.1% gelatin for 1 h and then washed 5x in 0.1% gelatin in PBS. Grids were floated on drops containing a secondary antibody (goat pAb to Ms IgG (6 nm gold) Abcam ab105285) diluted 1:8 in 0.1% gelatin in PBS for 30 min at room temperature, washed 5x in water, and then stained with 2% uranyl acetate, as previously described^57^. For DLB fibrils, 0.1% BSA was substituted for 0.1% gelatin.

### Transmission electron microscopy of DLB or MSA brain-derived fibrils and R8-LMNPs

Human MSA α-syn fibrils were mixed with R8-LMNPs (3 μg/mL) and incubated at 4 °C overnight. The next day 3 μL of the sample was spotted on a glow discharged carbon formvar 400 mesh grid and the sample was stained with 2% uranyl acetate, as described above. All grids were imaged at 6800x to 49,000x magnification on a FEI Tecnai Electron Microscope operating at 120 kV.

### Extraction of fibrils from AD brain tissue

Extraction of sarkosyl-insoluble fibrils from neuropathologically confirmed brain samples of patients diagnosed with AD was performed using the method previously described by Fitzpatrick et al. without any modifications^58^. The fibrils were confirmed to be a mixture of tau and amyloid-β by immunogold labeling with amyloid-β antibody, D54D2 (Cell Signaling Technology), and tau antibody, Tau-5 (Invitrogen).

### Immunogold labeling of AD brain-derived fibrils with amyloid-β antibody

Grids were glow discharged at 15 mA for 30 s using the PELCO easiGlow system. 3 μL of AD brain-extracted fibrils were spotted on the grids for 3 min. Grids were blotted and blocked in 0.1% BSA in PBS for 10 min, transferred to primary antibody (1:50 dilution of D54D2) for 1 h at room temp or to blocking buffer (no primary antibody control). Grids were washed 5x in blocking buffer, transferred to secondary (1:8 Goat pAb to Rabbit IgG (12 nm gold) (Abcam ab105298) for 30 min at room temperature. Grids were washed 5X in water and stained with 2% uranyl acetate.

### Double labeling of DLB, MSA, or AD brain-derived fibrils with α-syn antibody

Grids were glow discharged at 15 mA for 30 s with the PELCO easiGlow system. Brain-extracted fibrils were incubated with R8-LMNPs (10 μg/mL) for 4 h and then spotted on a grid for 4 min. Grids were then blocked with 0.1% gelatin in PBS for 10 minutes and transferred to a 1:100 dilution of LB509 diluted in 0.1% gelatin for 1 h. The grids were washed 5x in 0.1% gelatin in PBS and then transferred to the secondary antibody (goat pAb to Ms – 6 nm gold, Abcam ab105285) mixed with R8-LMNPs (15 μg/mL) for 30 min at room temperature. Grids were washed 5x with water and stained with 2% uranyl acetate. For DLB and AD fibrils, 0.1% BSA replaced 0.1% gelatin.

### Double labeling of AD brain-derived fibrils with amyloid-β antibody

Double labeling protocol is the same as for MSA fibrils, except 0.1% BSA in PBS was used in place of 0.1% gelatin in PBS for blocking and primary antibody was 1:50 dilution of D54D2 (Cell Signaling Technology, 8243).

### LMNP ELISA

LMNP ELISA was carried out in the same manner as the peptide ELISA, except rather than peptide, R8-LMNPs and 24mer-LMNPs were diluted in PBS and the plates were probed with primary antibody mouse anti-dextran antibody (Dx1, Stem Cell Technologies) at a 1:300 dilution. Monomer and fibril wells without LMNPs were probed with an anti-α-syn antibody (BD Transduction Labs 610787) as a surrogate loading control. LMNP concentration was confirmed using inductively coupled plasma mass spectrometry.

### Animal experiments

All animal experiments were approved by the UCLA Animal Research Committee and performed under oversight of the Division of Laboratory Animal Medicine (DLAM) under the IACUC protocol number ARC-2018-086. M83 mice (Jackson Laboratories: JAX:000664) were housed on a 12-h light–dark schedule. All live animal scans were approved and performed at the Functional Biological Imaging Core at the Zilkha Neurogenetic Institute (University of Southern California) under the IACUC protocol number 20658.

Heterozygous male and female M83 mice or wildtype controls were aged to 12–22 months. Mice were injected with R8-LMNPs or amine-MNP (10 mg/kg, i.v.) following intranasal administration of mannitol and then subjected to MR image acquisition (described below). Mice were euthanized (described below) and brain tissue was collected for transmission electron microscopy (described below), inductively coupled plasma mass spectrometry (described below) or immunohistochemical staining (described below).

### Euthanasia of mice

Mice were sacrificed by overdose with pentobarbital and then transcardial perfusion with perfusion buffer (1x PBS with sodium vanadate, leupeptin, aprotinin, pepstatin, sodium pyrophosphate, sodium fluoride, PMSF). For biochemical studies, the brain was removed and immediately frozen in liquid nitrogen and stored at −80 °C until used. For histological studies, the brain was removed and underwent three nights post-fixation in neutral buffered formalin (Thermo Fisher Scientific), transfer to 70% EtOH, and processing and embedding in paraffin. The blocks were then sectioned into 12-μm sections using a microtome (Leica Biosystems).

### Transmission electron microscopy and energy-dispersive X-ray spectroscopy of mouse brain tissue

Tissue from the brainstem, cerebellum, hippocampus, and cortex of each mouse brain was dissected, cut into small blocks, immediately fixed in 2.5% glutaraldehyde + 4% paraformaldehyde in 0.1 M sodium cacodylate buffer (pH 7.4), and further fixed with 1% osmium tetroxide in 0.1 M cacodylate buffered solution for 1 h. The specimens were then dehydrated in 30–100% ethanol. The tissue specimens were embedded in Epon-Araldite and polymerized at 60 °C for 48 h. The samples were cut into ultrathin sections with an ultramicrotome. The ultrathin sections were put on copper grids (200 mesh) and stained with uranyl acetate and lead citrate and then subjected to TEM observation using a JEOL 100CX transmission electron microscope with an AMT Camera System. Nanoparticle diameter was quantified using ImageJ. Energy-dispersive X-ray spectroscopy were carried out with a Titan 80–300 ST electron microscope (FEI Company) equipped with an extra-brightness field emission gun, an X-ray energy-dispersive detector (EDS), an electron energy filter and a charge-coupled device (CCD) camera.

### Inductively coupled plasma mass spectrometry of mouse brain tissue

The left hemisphere of each mouse brain was dried at 105 °C to constant weight; 1 mL of concentrated nitric acid and 0.2 mL of concentrated hydrochloric acid were added to the weighted sample (100 mg) in a tube and heated at 80 °C for 2 h. After digestion, the solution was diluted to 10 mL with deionized water. The iron contents of brain tissue were assessed by ICP-MS (NexION 2000, PerkinElmer).

### MRI acquisition

Data were acquired on a MR Solutions MRI 7 T system equipped with a 24 cm bore diameter and 600 mT/m maximum gradient strength, and a 20 mm internal diameter quadrature birdcage mouse head coil. After mice were anesthetized by 1–1.5% isoflurane in room air in an induction chamber (SomnoSuite^TM^), they were positioned on the scanner bed, maintained at a temperature of 36–37 °C, and secured to prevent motion with ear bars and a bite bar. Ophthalmic ointment was applied over the subject’s eyes and a pneumatic pillow was placed over the animal’s abdomen to monitor respiration during the scans (SA Instruments,Inc.). Mice were maintained on 1.5–2% isoflurane in 95% oxygen from an oxygen concentrator (PureLine OC4000), delivered via a nose cone. After the scan, animals were placed in a heated recovery chamber until they were ambulatory before returning them to their home cages.

A positioning gradient echo sequence was first acquired to prepare the slice stacks for the 2D multi-gradient echo (MGE) sequence. The MGE parameters were as follows: echo times (TEs) = 4, 8.48, 12.96, 17.44, 21.92, and 26.40 ms; repetition time (TR) = 1200 ms; slice thickness = 0.5 mm; field of view (FOV) = 20 mm × 20 mm; matrix size = 256 × 256; number of slices = 28; and number of averages (NA) = 1.

### MRI image analysis

*Quantitative parametric mapping*: Prior to parametric fitting, MGE images were preprocessed using Fiji (ImageJ)^59^ software with bias correction and denoising (2D median filter with radius = 0.5). Bias correction was performed by dividing each frame of the MGE image stack by its estimated bias field, which was generated using FSL FAST (FMRIB’s Automated Segmentation Tool)^60^. Then, T2* maps were generated through a pixel-by-pixel exponential fitting of the signal intensities across the different TE times, using the MATLAB Rocketship v.1.4 module^61^. All fits with an r^2^ > 0.6 were included. Using Fiji software, pixels with fits having r^2^ values < 0.6 were set to not-a-number (NaN) and were not included in the analysis. Additionally, brain regions were extracted by manually delineating brain outlines on each slice and outside brain regions were set to NaN. Regions of interests (ROI), such as the brainstem and the hippocampus, were manually delineated using the polygon tool in Fiji. R2* maps were generated from the T2* maps, using the relationship T2* = 1/R2*. Mean T2* and R2* values for each ROI, for each subject, were obtained. GraphPad Prism was used for planned comparisons between groups.

#### Difference maps by group

MGE DICOM data were converted to NIfTI format and then averaged over all echoes to obtain a mean T2* image for each mouse. R2* maps were generated from the T2* maps, using the relationship T2* = 1/R2*. Data were brain extracted using RATS^62^ and then entered into the ANTS pipeline for creation of an unbiased, mean deformation template using rigid, affine and non-linear registration^63^. The resultant transformations were applied to the raw, multi-echo data after which data were fit for R2* with a loglinear fit using Tedana^64^ (https://zenodo.org/records/7926293). Percentage change in R2* voxel-based maps were computed for each mouse at 48 and 120 h using the corresponding pre-injection data by: (post-injection-pre-injection)/pre-injection x 100.

### Immunohistochemistry of mouse brain tissue

Immunohistochemistry was performed on 10-µm-thick coronal sections Briefly, selected sections were deparaffinized, rehydrated, and steamed with a citric acid-based unmasking solution (Vector Laboratories, Burlingame, CA) for 40 min to enhance antigen detection. Endogenous peroxidases were quenched with 0.6% hydrogen peroxide in methanol for 30 min and sections were permeabilized with 0.3% Triton/TBS for 10 min. Sections were blocked in 3% bovine serum albumin (BSA)-TBS with 5% normal goat serum for 45 min at 37 °C. Rabbit polyclonal antibody against α-syn phosphorylated at S129 (pS129, EMD Millipore, Burlington, MA)) or monoclonal antibody against misfolded α-syn (5G4, EMD Millipore) was incubated at 37 °C for 1 h, and then overnight at 4 °C. After washing, sections were incubated with a biotinylated secondary antibody for 1 h at 37 °C and followed by avidin-biotin-peroxidase complex (ABC-HRP; Vector Laboratories, Burlingame, CA) for 1 h at 37 °C. Bound antibody complexes were visualized using a 3,3’-diaminobenzidine (DAB) Substrate Kit (Vector Laboratories, Burlingame, CA). All antibodies were diluted in 1X Tris-Buffered Saline with 0.1% Tween 20 (TBST). All slides contained a control section with substitution of TBST for the primary antibodies.

## Supplementary information

Supplementary information

## Data Availability

All data needed to evaluate the conclusions in the paper are present in the paper and/or the Supporting Information.
