# Supplementary material for: Liganded magnetic nanoparticles for magnetic resonance imaging of α-synuclein
Source: NPJ Parkinsons Dis. 2025 Apr 23;11:88. doi: 10.1038/s41531-025-00918-z (PMC12019173; doi:10.1038/s41531-025-00918-z)
Supplement: Supplementary file 1 — Supplementary information [file 41531_2025_918_MOESM1_ESM.pdf]

Liganded magnetic nanoparticles for magnetic resonance imaging of α-synuclein
Supporting Information

Hope Pan[1*], Melinda Balbirnie[1*], Ke Hou[1], Naomi S. Sta Maria[2], Shruti Sahay[1], Paul Denver[3,4], Stefano Lepore[5], Mychica Jones[3,4], Xiaohong Zuo[3.4], Chunni Zhu[4,6], Hilda Mirbaha[7], Hedieh Shahpasand-Kroner[3,4], Marisa Mekkittikul[3,4], Jiahui Lu[1], Carolyn J Hu[1], Xinyi Cheng[1], Romany Abskharon[1], Michael R. Sawaya[1], Christopher K. Williams[4,7], Harry V. Vinters[4,7], Russell E. Jacobs[2], Neil G. Harris[5], Gregory M. Cole[3,4], Sally A. Frautschy[3,4], David S. Eisenberg[1**]

[1]Department of Chemistry and Biochemistry, Department of Biological Chemistry, UCLA-DOE Institute, Molecular Biology Institute, UCLA, Los Angeles CA, 90095; [2]Department of Research Physiology, Department of Neuroscience, Keck School of Medicine at USC, Los Angeles, CA 90033; [3]Geriatric Research Education and Clinical Center, Greater Los Angeles Veterans Affairs Healthcare System, West Los Angeles VA Medical Center, Los Angeles, CA, 90073; [4]Department of Neurology, David Geffen School of Medicine at UCLA, Los Angeles, CA, 90095; [5]Department of Neurosurgery, David Geffen School of Medicine at UCLA, Los Angeles, CA 90095; [6]Brain Research Institute Electron Microscopy Core Facility, David Geffen School of Medicine, UCLA, Los Angeles, CA 90095; [7]Department of Pathology and Laboratory Medicine, David Geffen School of Medicine, UCLA, Los Angeles, CA 90095

*These authors contributed equally

**To whom correspondence should be addressed: David S. Eisenberg: University of California-Los Angeles 611 Charles E Young Drive, Boyer 201, Los Angeles, CA 90095; david@mbi.ucla.edu; Tel. (310) 825-3754, Fax. (310) 206-3914

Patient	Braak	Age	Sex	Region
DLB	N/A	90	F	Frontal
MSA	N/A	65	F	Cerebellum
AD	VI	86	F	Left temporal

Supplementary Table 1. Pathology information for patient-derived samples.

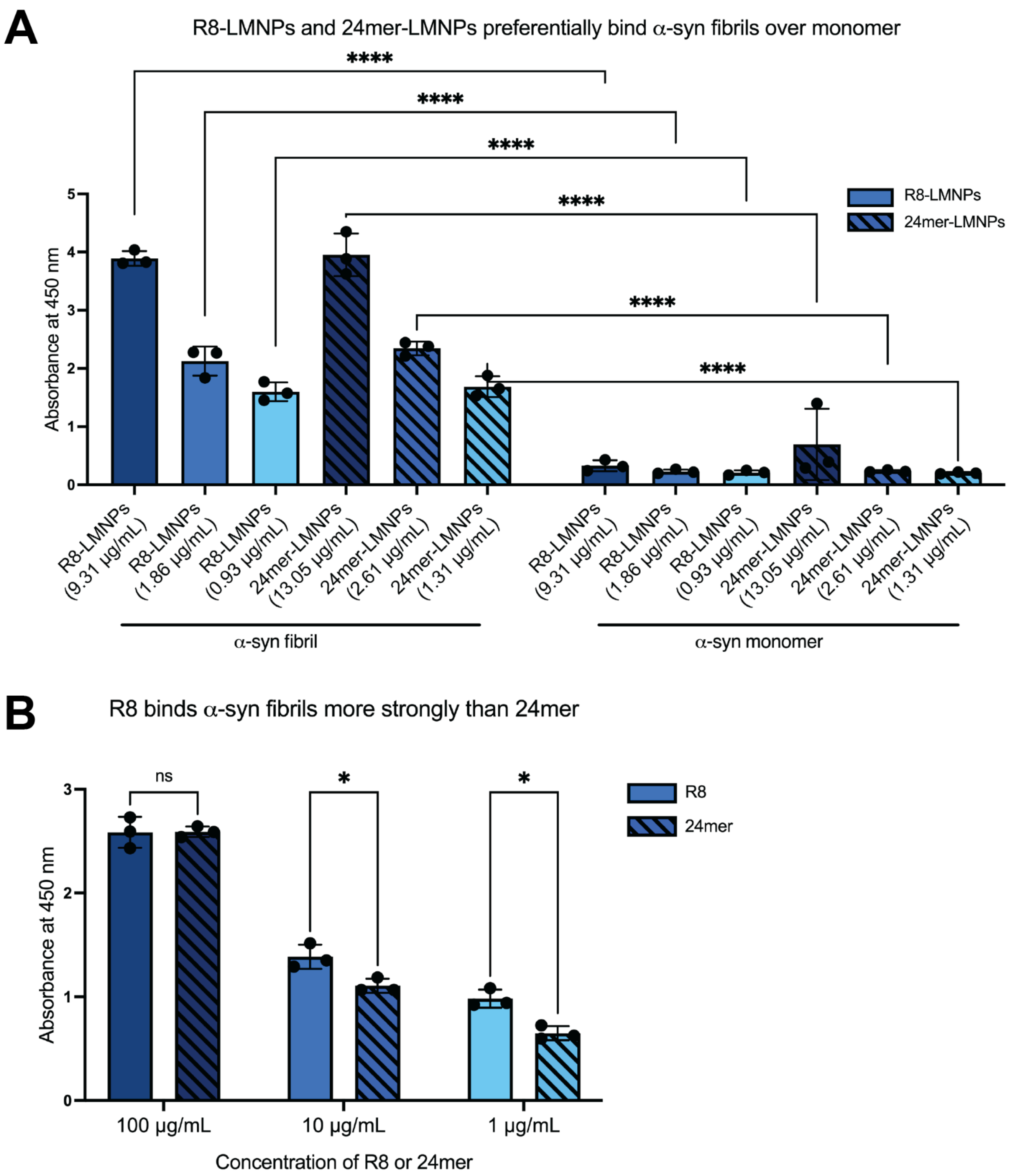

Supplementary Figure 1. R8 binds recombinant α-syn fibrils. (A) ELISA assesses binding of R8 and 24mer to α-syn fibril (left) and monomer (right). Both R8 (solid bars) and 24mer (striped bars) preferentially bind α-syn fibril over monomer. Statistical analysis was performed using two-way ANOVA (multiple comparisons using Šídák's multiple comparisons test; ns, $p > 0.05$; *, $p < 0.05$; **, $p < 0.01$; ***, $p < 0.001$; ****, $p < 0.0001$) in GraphPad Prism. **(B)** From the same ELISA in (A), comparison of R8 and 24mer binding to α-syn fibril. Statistical analysis was performed using multiple unpaired t-tests (multiple comparisons using Holm-Šídák method; ns, $p > 0.05$; *, $p < 0.05$) in GraphPad Prism.

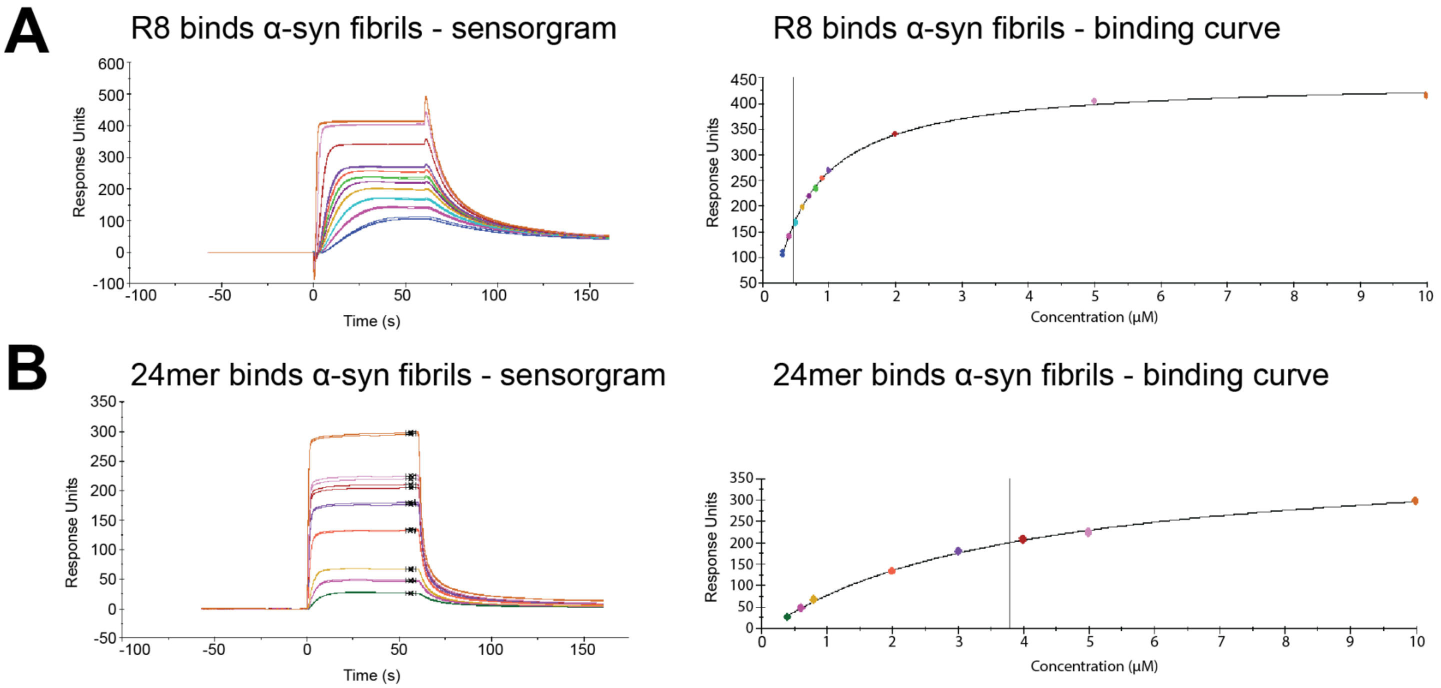

Supplementary Figure 2. R8 binds recombinant α-syn fibrils. To assess α-syn fibril binding by surface plasmon resonance (SPR), we immobilized α-syn fibrils on a CM5 SPR chip and measured affinities of **(A)** R8 and **(B)** 24mer. SPR measurements showed an increase in SPR signal (response units) with increase in inhibitor concentration for both peptides (left panels). The equilibrium dissociation constant (K_d) was calculated by steady state analysis (right panels). The apparent K_d for R8 was determined to be 0.47 μM, and the apparent K_d for 24mer was determined to be 3.7 μM under identical experimental conditions.

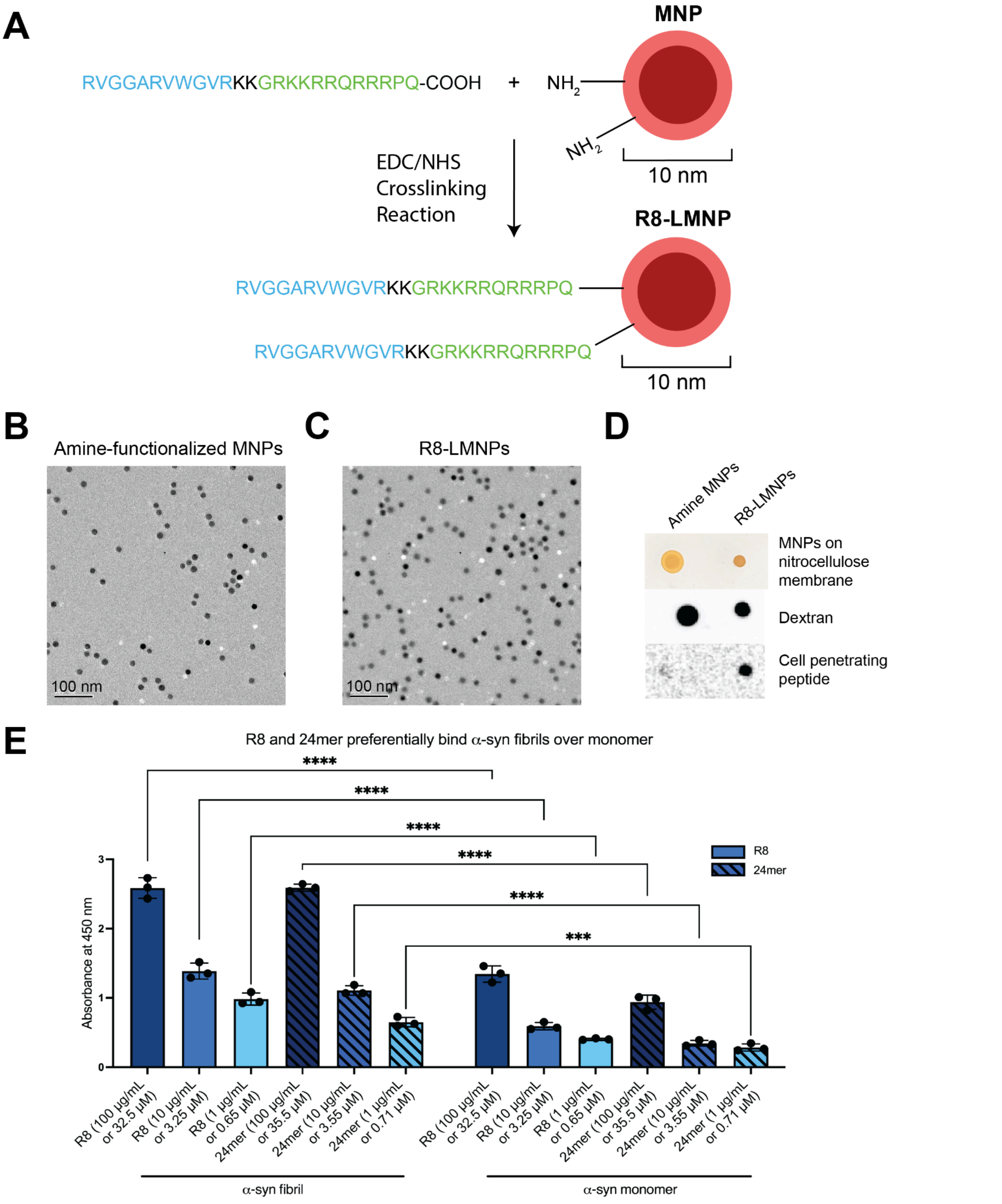

Supplementary Figure 3. R8-LMNPs bind recombinant α-syn fibrils. (A) R8-liganded magnetic nanoparticles (R8-LMNPs) are composed of a 10 nm diameter iron oxide core (brown) coated with dextran (pink), to which we covalently coupled the R8 peptide using EDC/NHS crosslinking chemistry. **(B)** Before conjugation, amine-functionalized nanoparticles are homogenous and well-dispersed. **(C)** After conjugation,

R8-LMNPs are still homogenous and well-dispersed. **(D)** 1.5 μL of both amine-functionalized MNPs and R8-LMNPs were blotted onto a nitrocellulose membrane. R8-LMNPs spread less far on the nitrocellulose membrane, indicating a change in functionalization of the nanoparticle. The membrane was probed with an antibody for dextran, which bound both amine-functionalized MNPs and R8-LMNPs, and an antibody for the cell penetrating peptide, which bound only R8-LMNPs. **(E)** ELISA assesses binding of R8-LMNPs and 24mer-LMNPs to α-syn fibril (left) and monomer (right). Both R8-LMNPs (solid bars) and 24mer-LMNPs (striped bars) preferentially bind α-syn fibril over monomer. Statistical analysis was performed using two-way ANOVA (multiple comparisons using Šídák's multiple comparisons test; ns, $p > 0.05$; *, $p < 0.05$; **, $p < 0.01$; ***, $p < 0.001$; ****, $p < 0.0001$) in GraphPad Prism.

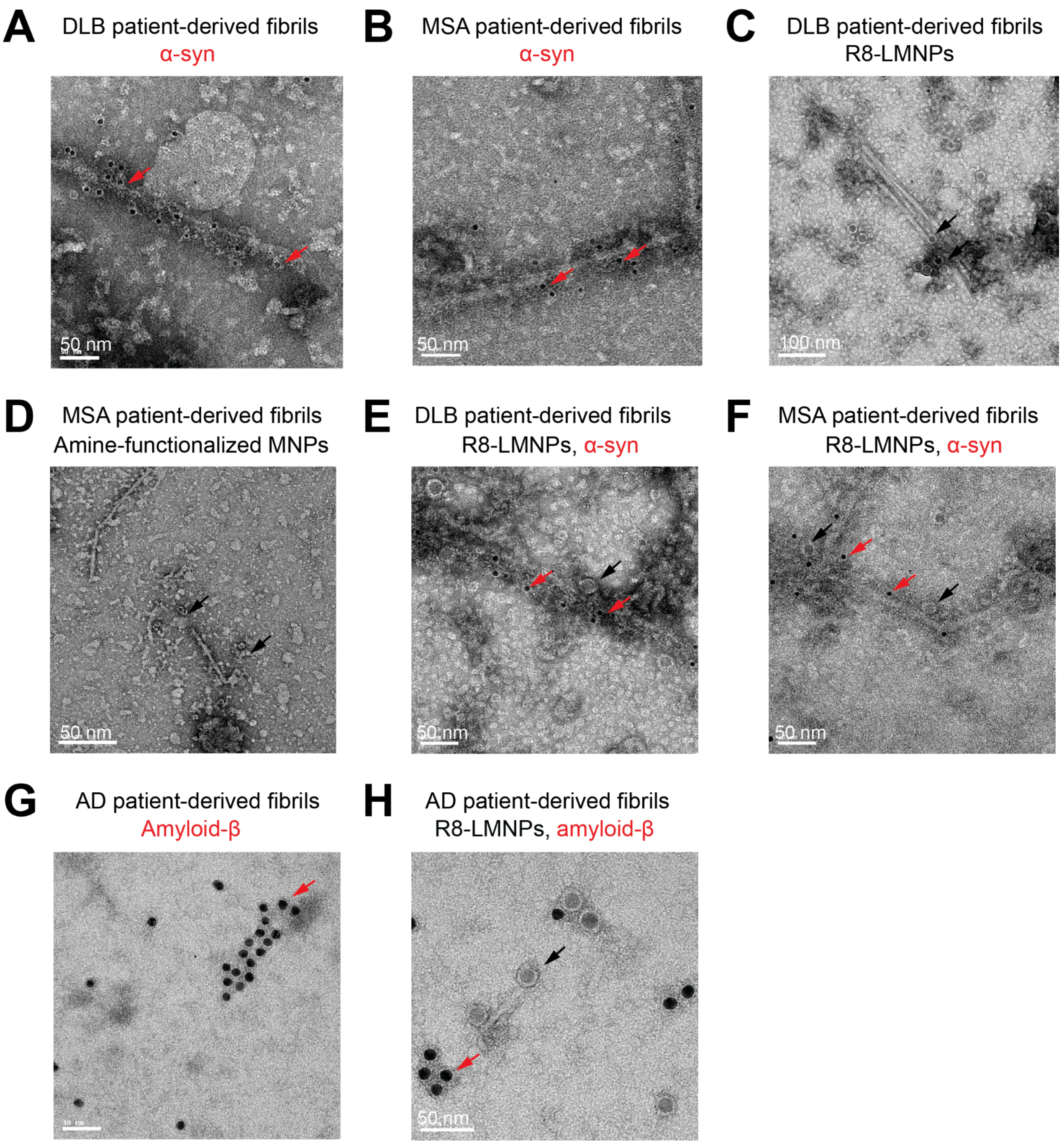

Supplementary Figure 4. R8-LMNPs bind recombinant, DLB brain-derived, and MSA brain-derived α-syn fibrils. (A-B) α-syn antibody LB509 (and secondary antibody conjugated to 6 nm gold, red arrows) strongly labeled **(A)** DLB brain-derived fibrils and **(B)** MSA brain-derived fibrils, indicating that they are α-syn fibrils. **(C)** R8-LMNPs labeled 10 nm DLB brain-derived fibrils more modestly than 5 nm DLB brain-derived fibrils shown in Figure 2D. **(D)** Unconjugated, amine-functionalized nanoparticles (black arrows) did not bind MSA brain-derived fibrils. **(E)** R8-LMNPs (10 nm, black arrows) and α-syn antibody LB509 (6 nm, red arrows) simultaneously bind DLB brain-derived fibrils. **(F)** R8-LMNPs (10 nm, black arrows) and α-syn antibody LB509 (6 nm, red arrows) simultaneously bind MSA brain-derived fibrils. **(G)** Amyloid-β antibody D54D2 (12 nm, red arrows) strongly labeled some AD brain-derived fibrils, indicating those fibrils were composed of amyloid-β. **(H)** R8-LMNPs (10 nm, black arrows) and amyloid-β antibody D54D2 (12 nm, red arrows) simultaneously bind some AD brain-derived fibrils.

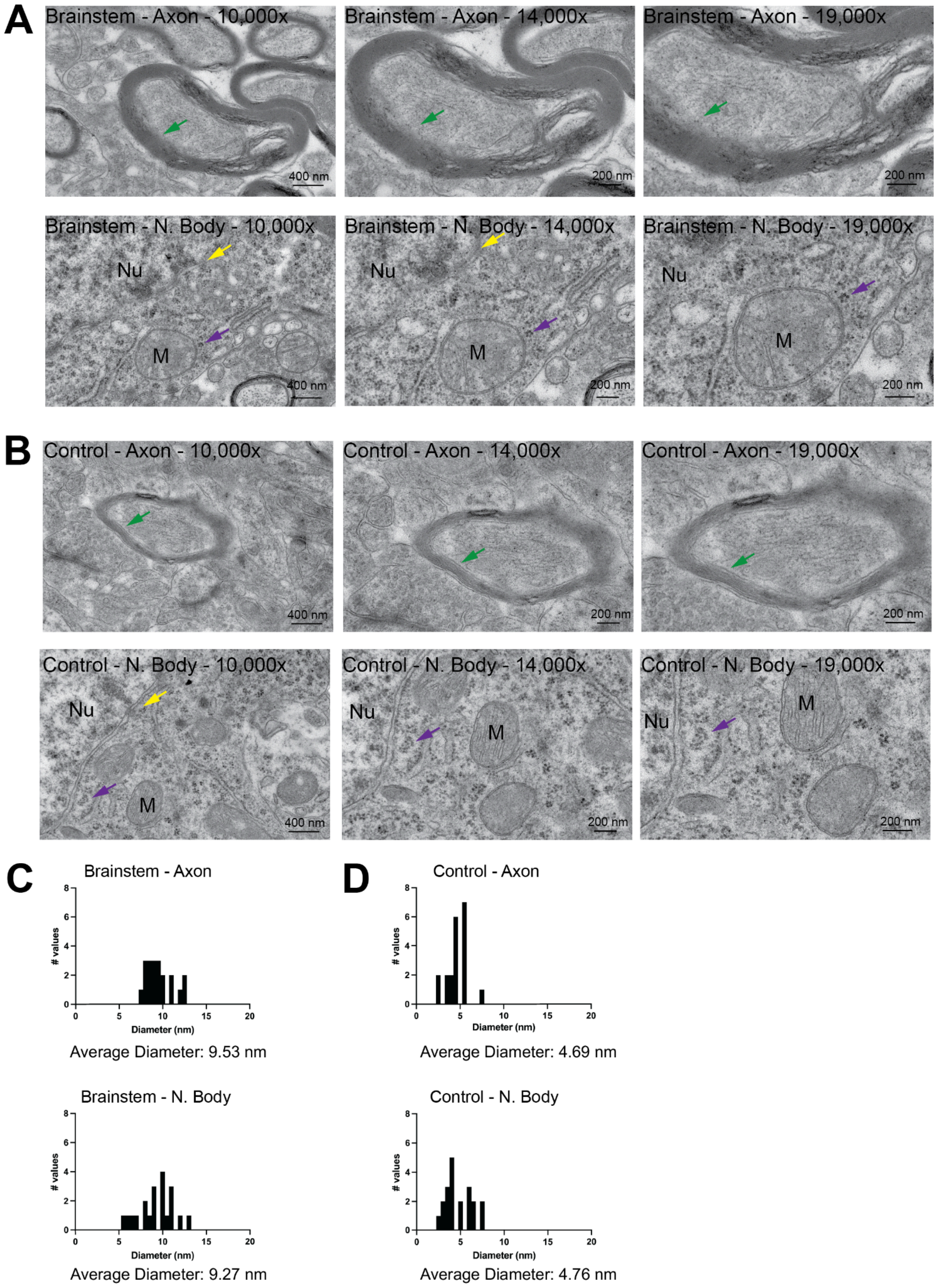

Supplementary Figure 5. Large electron-dense spots are visible in the brains of M83 mice treated with R8-LMNPs and not in control mice. (A) R8-LMNPs (10 mg/kg) were administered to aged M83 mice following intranasal administration of mannitol, and mice were euthanized six hours after administration. **(B)**

Mice that did not receive R8-LMNPs were euthanized and their tissue was used as a control. **(A-B)** Images of ultrathin sections of the brainstem were acquired at 10,000x, 14,000x, and 19,000x magnification. Axons are identified by their electron-dense myelin sheath (green arrow). Neuron cell bodies are identified by their large, vacuous nucleus surrounded by a double membrane (yellow arrow) and the presence of abundant ribosomes in their cytoplasm (purple arrow). Mitochondria are labeled with M and nuclei with Nu. **(C-D)** Distribution (n = 20) of the diameter of electron-dense spots in the tissue was measured in (**C**) aged M83 mice that received R8-LMNPs and (**D**) control mice.

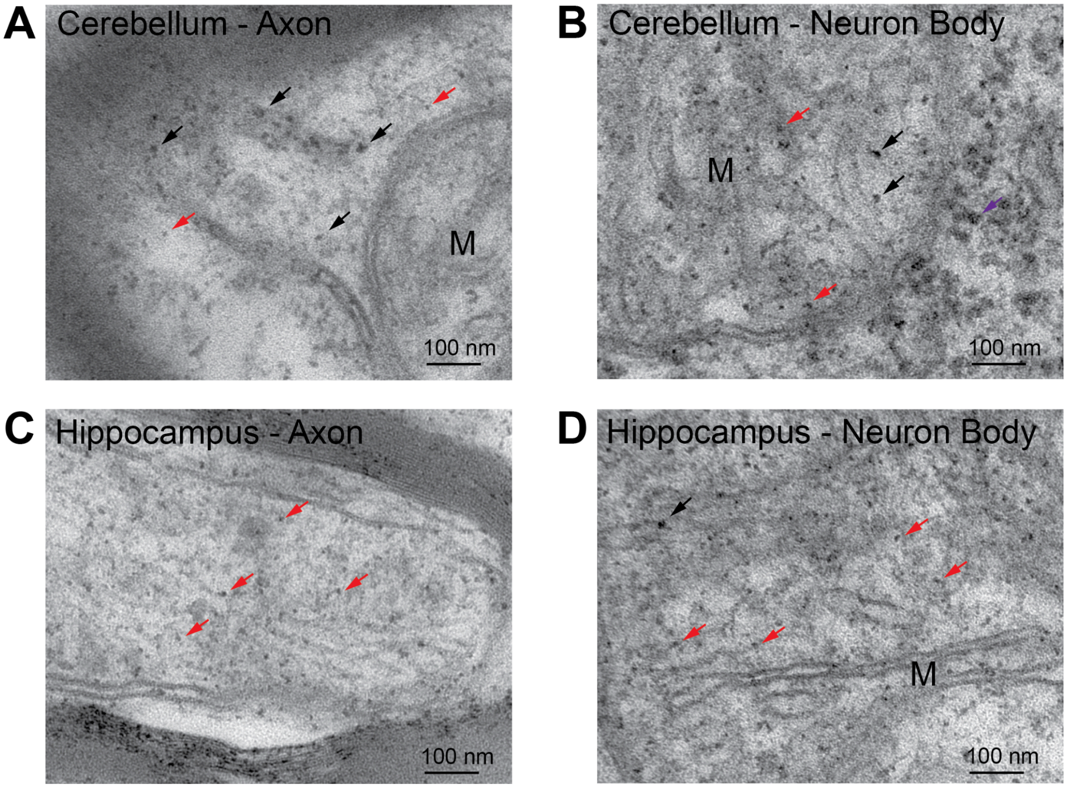

Supplementary Figure 6. Distribution of the diameter of electron-dense spots varies between brain regions. R8-LMNPs (10 mg/kg) were administered to aged M83 mice following intranasal administration of mannitol, and mice were euthanized six hours after administration. Images of ultrathin sections of the (**A-B**) cerebellum and (**C-D**) hippocampus were acquired using transmission electron microscopy. Electron-dense spots larger than 10 nm in diameter (black arrows) and smaller than 10 nm in diameter (red arrows) are visible in all three regions around axons (**A, C**) and neuron cell bodies (**B, D**). These electron-dense spots can be distinguished from ribosomes (purple arrows) in the cytoplasm of the cell body because ribosomes are clustered and about 30 nm in size. Mitochondria are labeled with M.

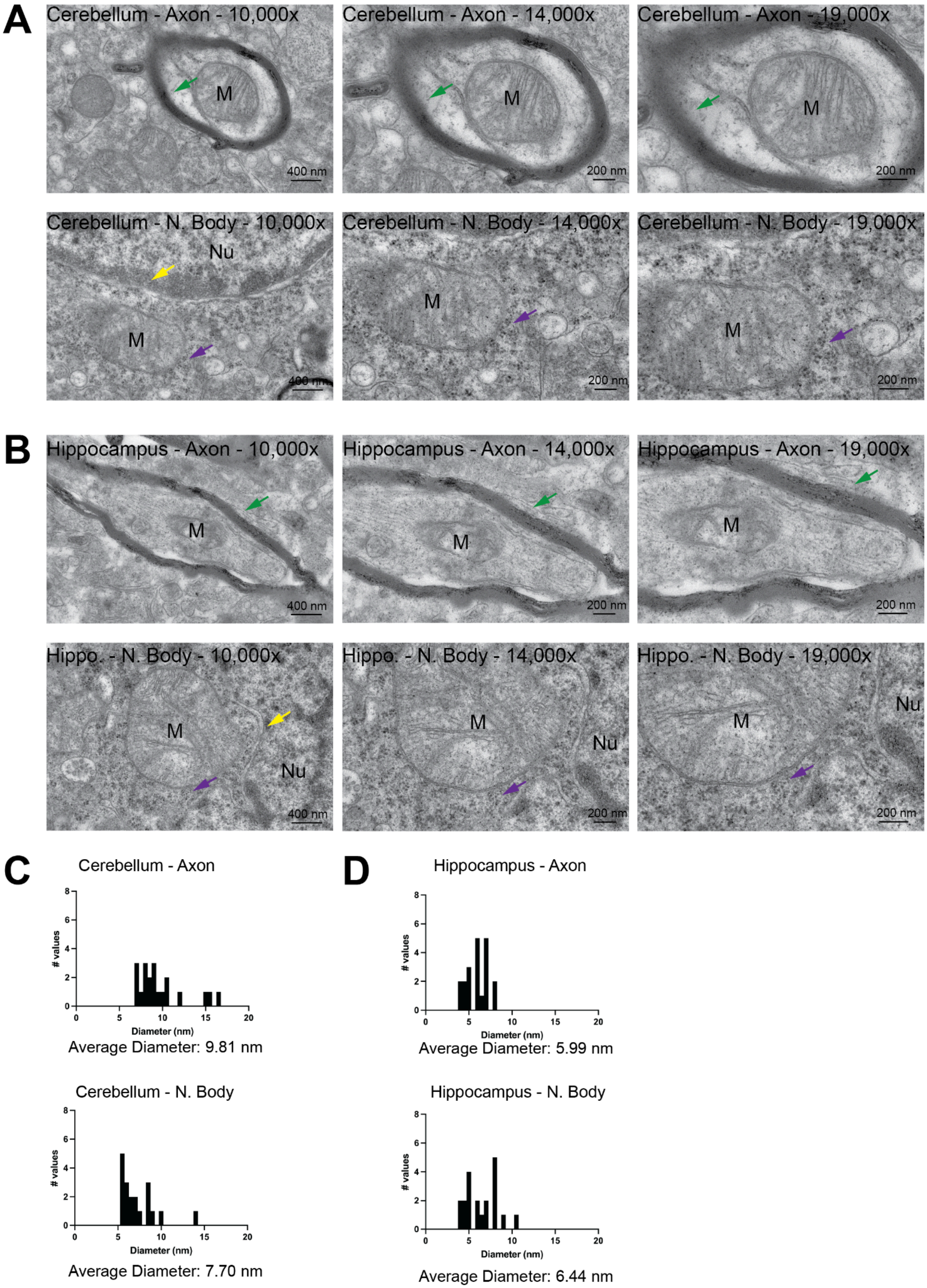

Supplementary Figure 7. Distribution of the diameter of electron-dense spots varies between brain regions. R8-LMNPs (10 mg/kg) were administered to aged M83 mice following intranasal administration of mannitol, and mice were euthanized six hours after administration. **(A-B)** Images of ultrathin sections of the (**A**)

cerebellum and (**B**) hippocampus were acquired at 10,000x, 14,000x, and 19,000x magnification. Axons are identified by their electron-dense myelin sheath (green arrow). Neuron cell bodies are identified by their large, vacuous nucleus surrounded by a double membrane (yellow arrow) and the presence of abundant ribosomes in their cytoplasm (purple arrow). Mitochondria are labeled with M and nuclei with Nu. **(C-D)** Distribution (n = 20) of the diameter of electron-dense spots in the tissue was measured in (**C**) cerebellum and (**D**) hippocampus.

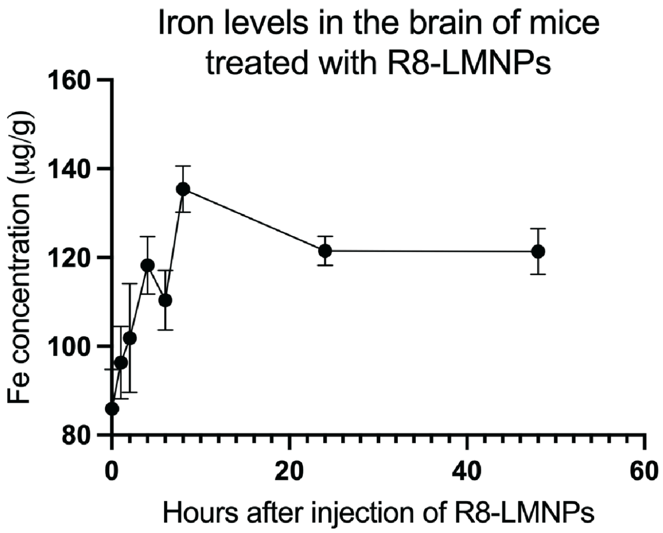

Supplementary Figure 8. R8-LMNPs remain in brains of M83 mice after 48 hours. R8-LMNPs (10 mg/kg) were administered to aged M83 mice, and mice were euthanized at various times up to 48 hours after administration (n = 3 mice per time point). Brains from aged M83 mice that did not receive R8-LMNPs were used as a 0 hour or "baseline" time point. From one to eight hours after administration of R8-LMNPs, iron levels increased in the brains of M83 mice. At 24 hours and 48 hours after administration of R8-LMNPs, iron levels plateaued but were still higher than those at baseline.

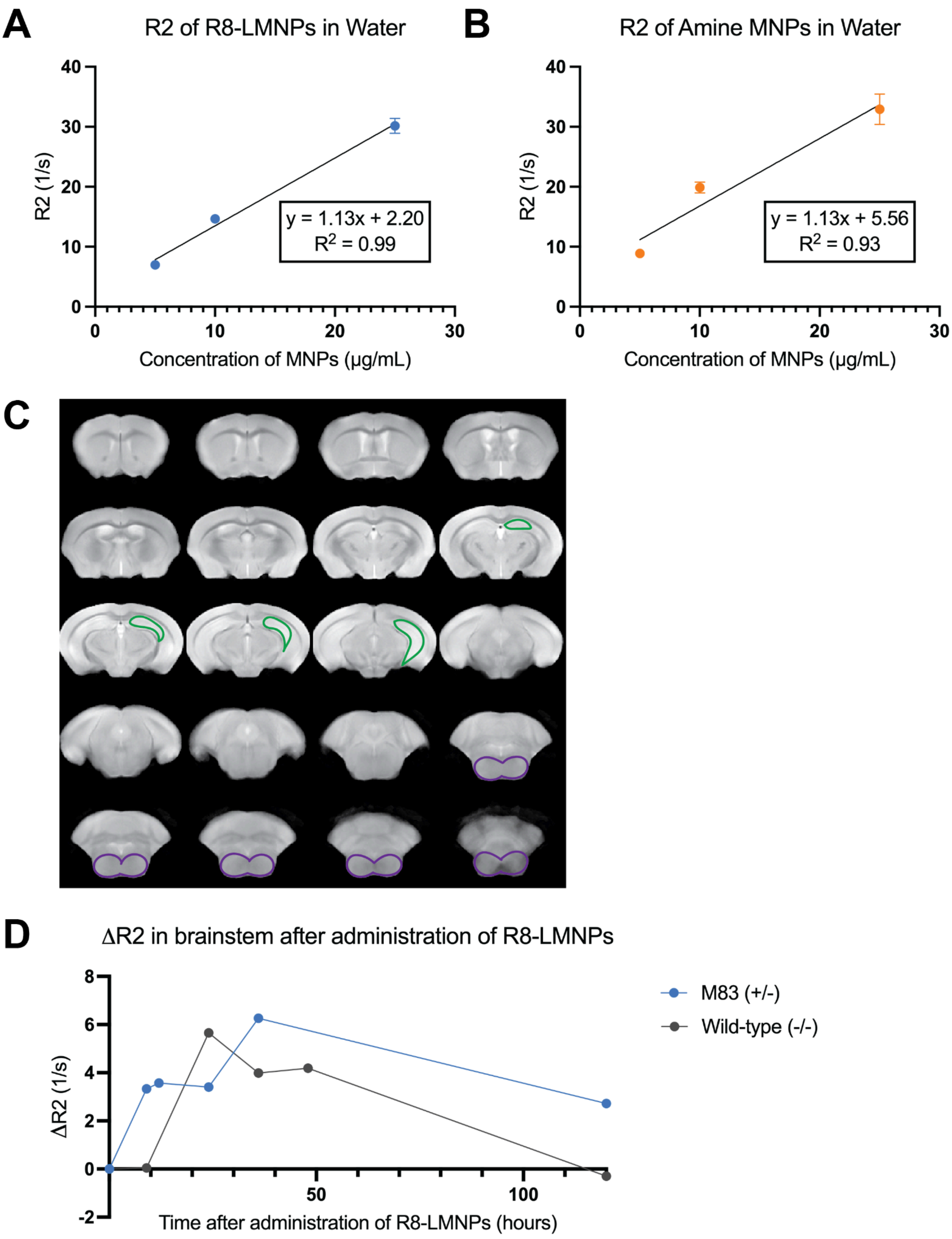

Supplementary Figure 9. R8-LMNPs can be used as an MRI contrast agent to distinguish M83 mice from age-matched, wild-type controls. (A-B) R2 relaxation rates of **(A)** R8-LMNPs and **(B)** amine-functionalized MNPs in water. Within the range of 5 to 25 µg/mL, R8-LMNPs and amine-functionalized MNPs cause a linear increase in R2. **(C)** Grayscale maps display 20 anterior to posterior coronal sections of the brain. Regions of interest are highlighted: brainstem (purple) and hippocampus (green). **(D)** Average R2 relaxation rate measured in the brainstem of one aged M83 mouse (blue) and one age-matched wild-type control mouse (gray) that received R8-LMNPs (10 mg/kg) over the course of 120 hours.

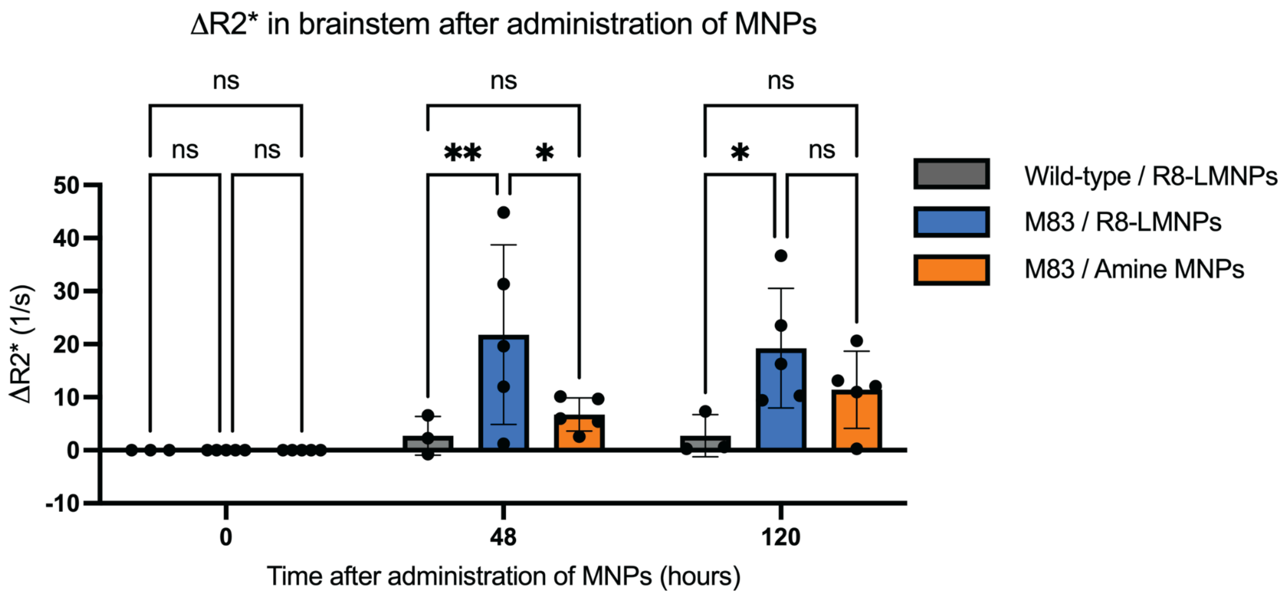

Supplementary Figure 10. R8-LMNPs can be used as an MRI contrast agent to distinguish M83 mice from age-matched, wild-type controls. Average change in R2* relaxation rate in the brainstem of M83 mice that received R8-LMNPs (n = 5, blue), wild-type control mice that received R8-LMNPs (n = 3, gray), and M83 mice that received amine-functionalized MNPs (n = 5, orange) either 0, 48, or 120 hours after administration of R8-LMNPs or amine MNPs. Statistical analysis was performed using two-way ANOVA (multiple comparisons using Šídák's multiple comparisons test; ns, $p > 0.05$; *, $p < 0.05$; **, $p < 0.01$; ***, $p < 0.001$; ****, $p < 0.0001$) in GraphPad Prism.

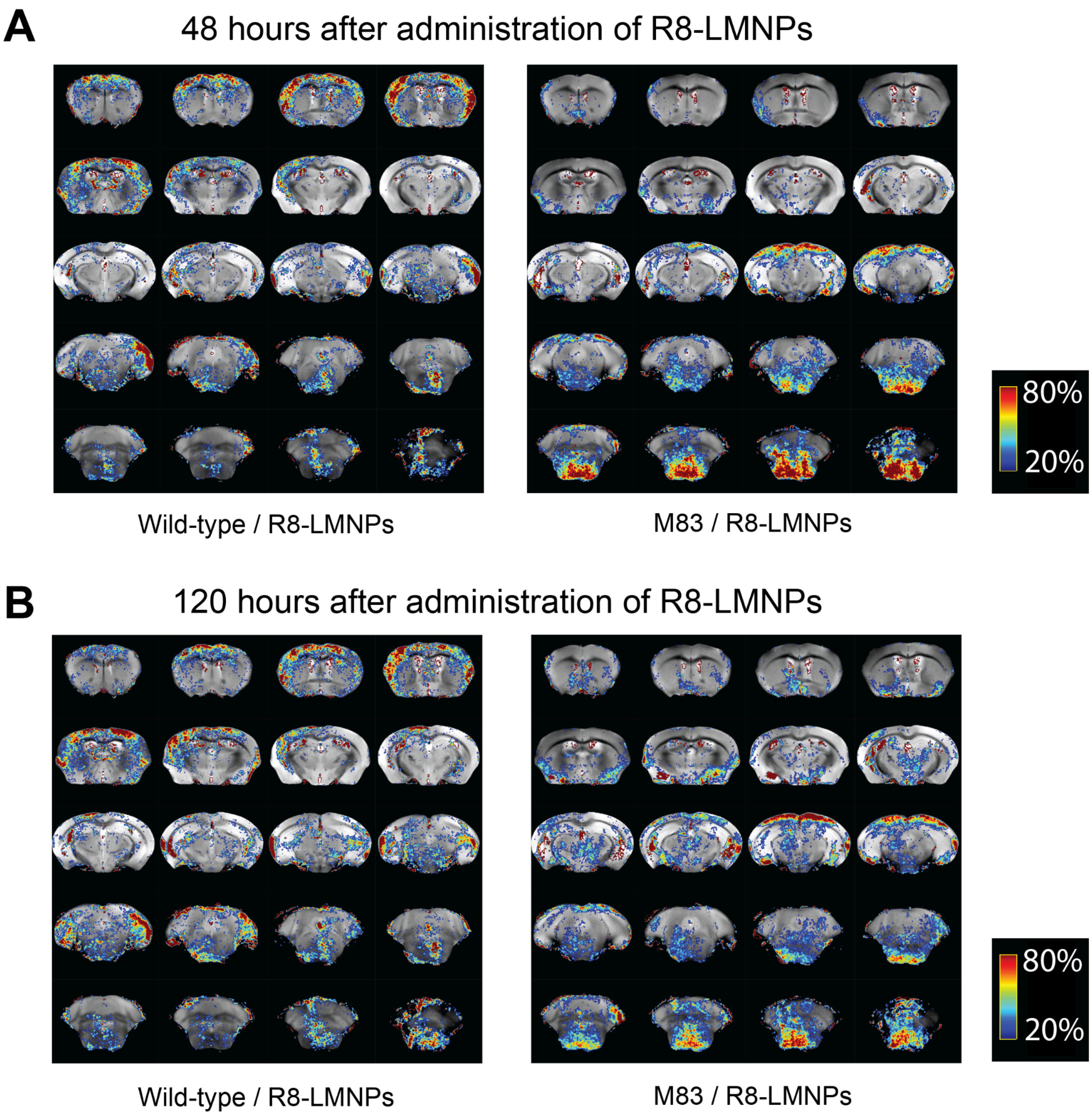

Supplementary Figure 11. R2* % difference maps of the brains of M83 mice and age-matched wild-type control mice after injection of R8-LMNPs. (A) 48 hours after injection, and **(B)** 120 hours after injection. R2* % difference maps display 20 anterior to posterior coronal sections of the brain. They are colorized on a scale from 0 to 100 ms, with cooler colors representing smaller % increase in R2* and warmer colors representing larger % increase in R2*. Each map represents the average % increase in R2* of all mice in a group.

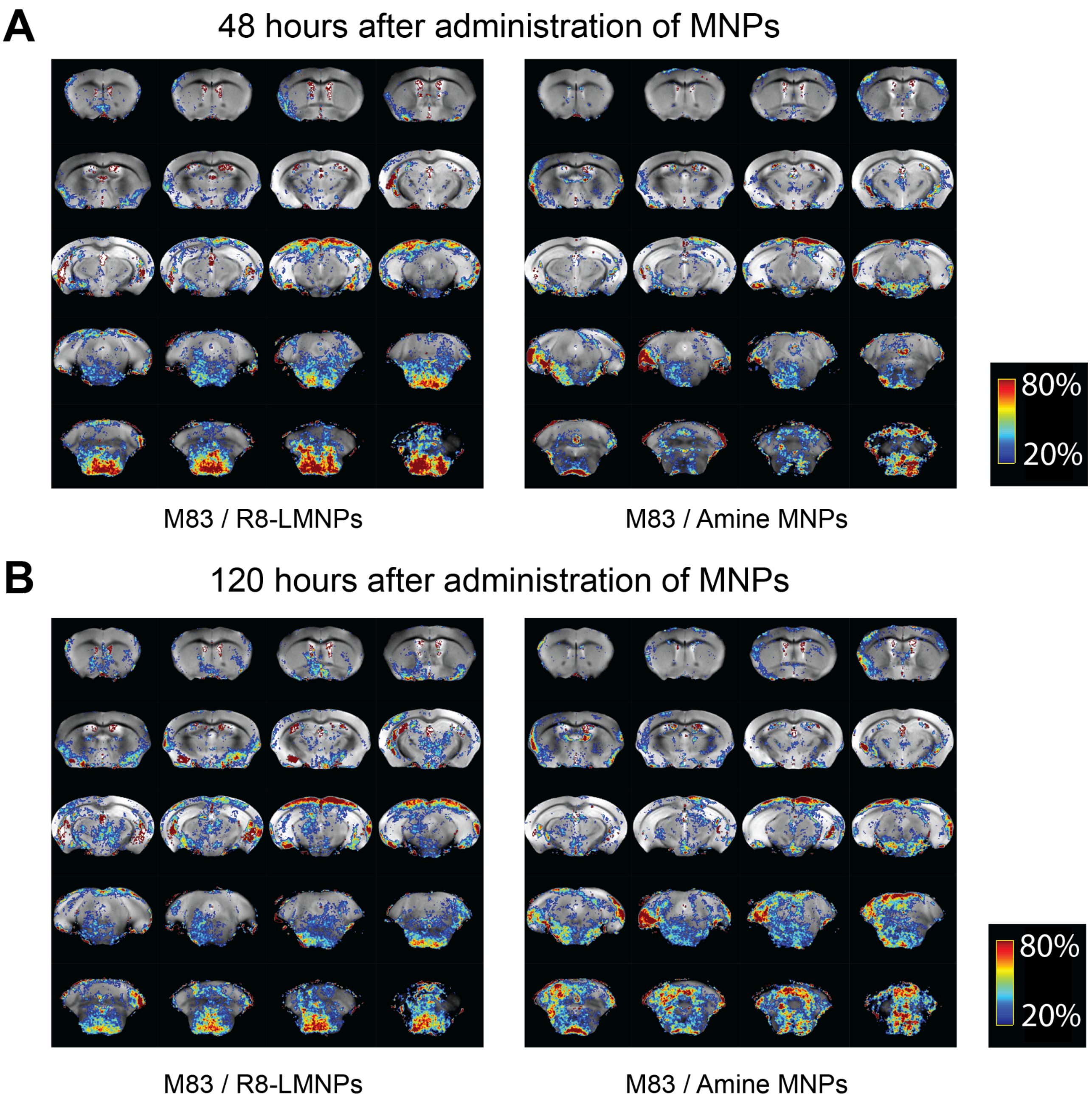

Supplementary Figure 12. R2* % difference maps of the brains of M83 mice after injection of R8-LMNPs or amine-functionalized MNPs. (A) 48 hours after injection, and **(B)** 120 hours after injection. R2* % difference maps display 20 anterior to posterior coronal sections of the brain. They are colorized on a scale from 0 to 100 ms, with cooler colors representing smaller % increase in R2* and warmer colors representing larger % increase in R2*. Each map represents the average % increase in R2* of all mice in a group.

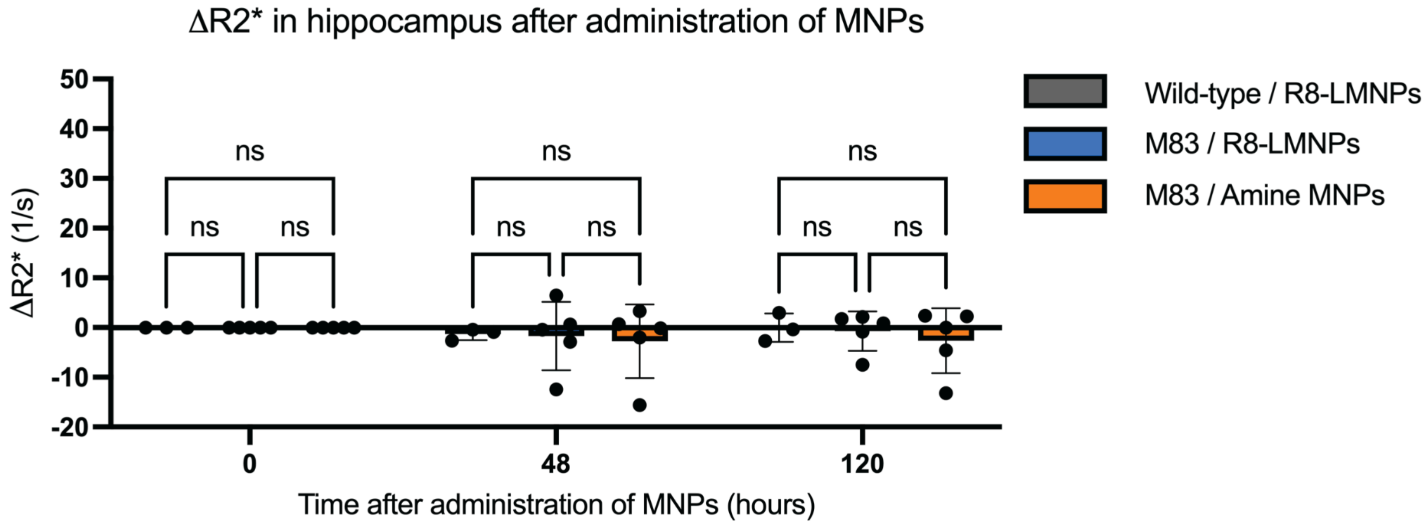

Supplementary Figure 13. Differences in R2* relaxation rates caused by R8-LMNPs are specific to regions of the brain with α-syn pathology. Average change in R2* relaxation rate in the hippocampus of M83 mice that received R8-LMNPs (n = 5, blue), wild-type control mice that received R8-LMNPs (n = 3, gray), and M83 mice that received amine-functionalized MNPs (n = 5, orange) either 0, 48, or 120 hours after administration of R8-LMNPs or amine MNPs. Statistical analysis was performed using two-way ANOVA (multiple comparisons using Šídák's multiple comparisons test; ns, $p > 0.05$; *, $p < 0.05$; **, $p < 0.01$; ***, $p < 0.001$; ****, $p < 0.0001$) in GraphPad Prism.

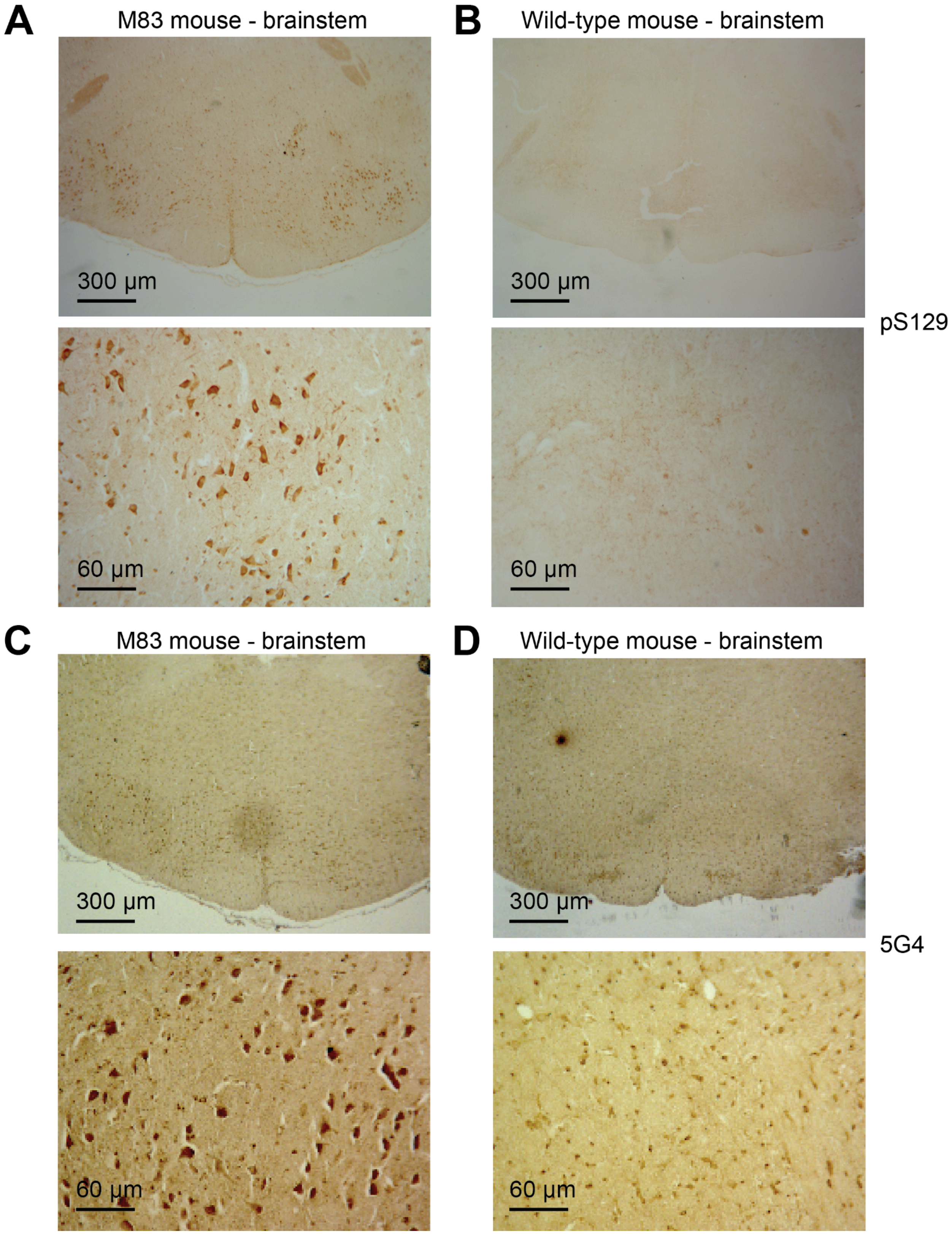

Supplementary Figure 14. Aged M83 mice have abundant α-syn pathology in the brainstem. Following MR imaging, 22-month-old, female, heterozygous M83 mice and age-matched, wild-type control mice were euthanized via transcardiac perfusion, and their brain tissue was collected and fixed. Sections of fixed brain tissue were stained using **(A-B)** a rabbit polyclonal antibody against α-syn phosphorylated at S129 (pS129) and **(C-D)** a monoclonal antibody against misfolded α-syn (5G4). **(A, C)** There is α-syn pathology in the brainstem of M83 mice. **(B, D)** There is no α-syn pathology in the brainstem of wild-type control mice.

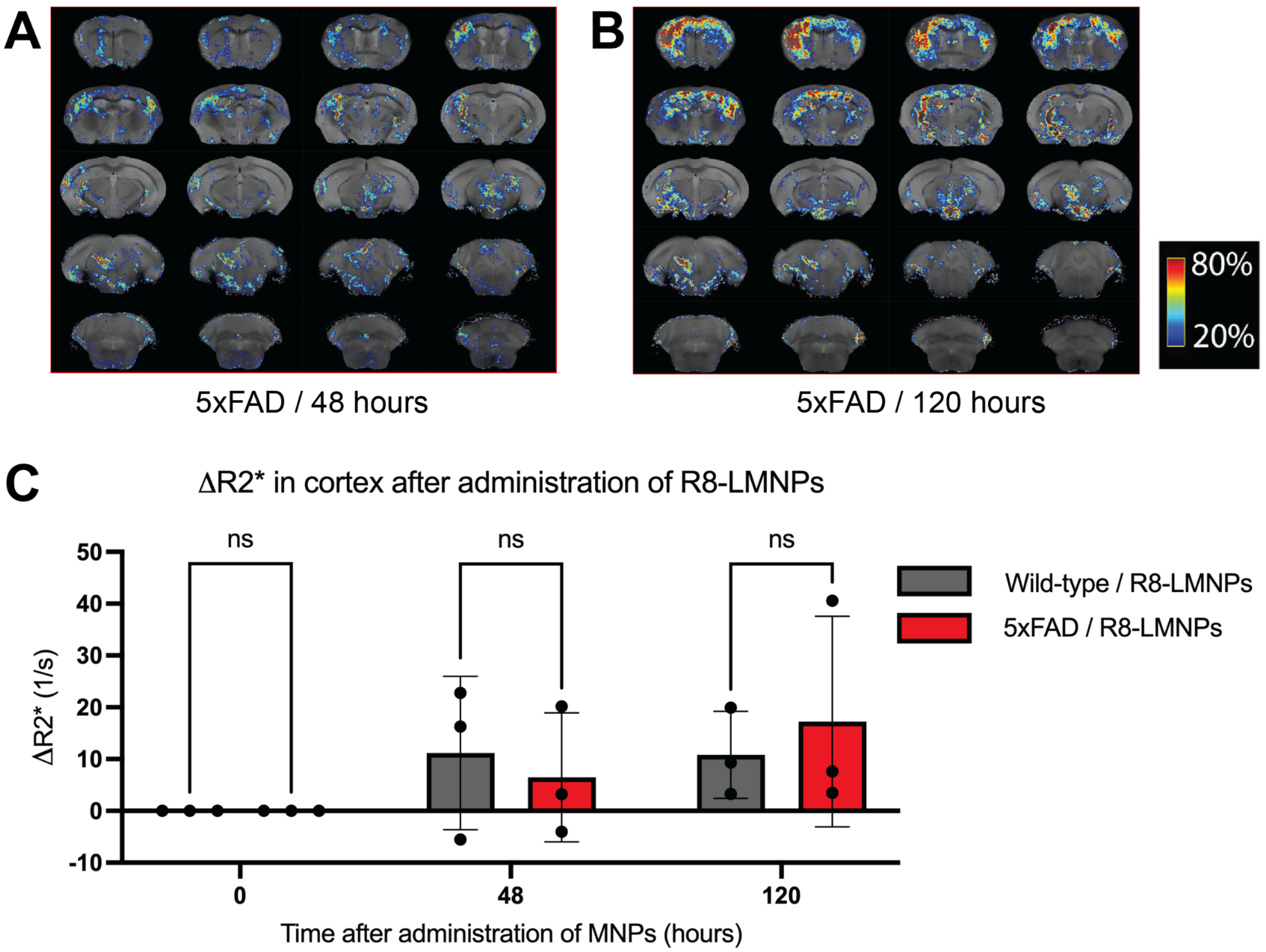

Supplementary Figure 15. R8-LMNPs do not distinguish 5xFAD mice from wild-type control mice. (A-B) Average R2* % difference maps of the brains of 5xFAD mice **(A)** 48 or **(B)** 120 hours after injection of R8-LMNPs. R2* % difference maps display 20 anterior to posterior coronal sections of the brain. R2* % difference maps are colorized on a scale from 0 to 100 ms, with cooler colors representing smaller % increase in R2* and warmer colors representing larger % increase in R2*. **(C)** Average change in R2* relaxation rate in the cortex of 5xFAD mice (n = 3, red) and wild-type control mice (n = 3, gray) that received R8-LMNPs (n = 3, gray) either 0, 48, or 120 hours after administration of R8-LMNPs. Statistical analysis was performed using two-way ANOVA (multiple comparisons using Šídák's multiple comparisons test; ns, $p > 0.05$; *, $p < 0.05$; **, $p < 0.01$; ***, $p < 0.001$; ****, $p < 0.0001$) in GraphPad Prism.

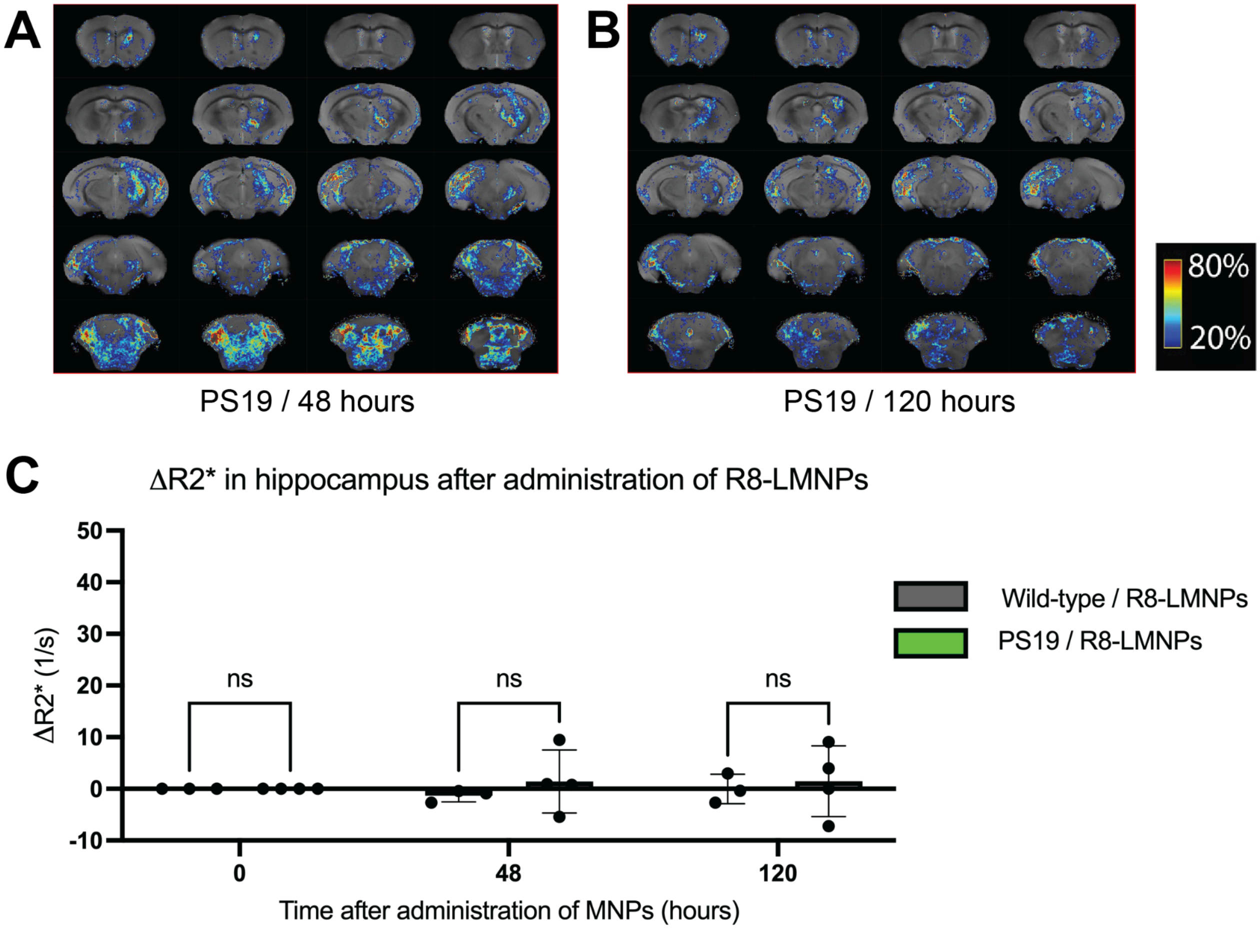

Supplementary Figure 16. R8-LMNPs do not distinguish PS19 mice from wild-type control mice. (A-B) Average R2* % difference maps of the brains of AD fibril-seeded PS19 mice **(A)** 48 or **(B)** 120 hours after injection of R8-LMNPs. R2* % difference maps display 20 anterior to posterior coronal sections of the brain. R2* % difference maps are colorized on a scale from 0 to 100 ms, with cooler colors representing smaller % increase in R2* and warmer colors representing larger % increase in R2*. **(C)** Average change in R2* relaxation rate in the hippocampus of PS19 mice (n = 4, green) and wild-type control mice (n = 3, gray) that received R8-LMNPs (n = 3, gray) either 0, 48, or 120 hours after administration of R8-LMNPs. Statistical analysis was performed using two-way ANOVA (multiple comparisons using Šídák's multiple comparisons test; ns, $p > 0.05$; *, $p < 0.05$; **, $p < 0.01$; ***, $p < 0.001$; ****, $p < 0.0001$) in GraphPad Prism.
